# Insights into the bioluminescence systems of three sea pens (Cnidaria: Anthozoa): from de novo transcriptome analyses to biochemical assays

**DOI:** 10.1098/rsob.240262

**Published:** 2025-04-30

**Authors:** Laurent Duchatelet, Gabriela A. Galeazzo, Constance Coubris, Laure Bridoux, René Rezsohazy, Marcelo R. S. Melo, Martin Marek, Danilo T. Amaral, Sam Dupont, Anderson Garbuglio de Oliveira, Jérôme Delroisse

**Affiliations:** ^1^ Earth and Life Institute, Université catholique de Louvain, Louvain-la-Neuve, Walloon Brabant, Belgium; ^2^ Departamento de Oceanografia Física, Química e Geológica, Universidade de São Paulo, Sao Paulo, Brazil; ^3^ Louvain Institute of Biomolecular Science and Technology, Université catholique de Louvain, Louvain-la-Neuve, Walloon Brabant, Belgium; ^4^ Departamento de Oceanografia Biológica, Universidade de São Paulo, Sao Paulo, Brazil; ^5^ International Clinical Research Center, Masaryk University, Brno, Czech Republic; ^6^ Centre for Natural and Human Sciences, Universidade Federal do ABC, Santo Andre, Brazil; ^7^ Biological and Environmental Sciences, Goteborgs Universitet, Goteborg, Sweden; ^8^ International Atomic Energy Agency Marine Environment Laboratories, Monaco, Monaco; ^9^ Department of Chemistry and Biochemistry, Yeshiva University, New York, NY, USA; ^10^ Marine Organisms and Biomimetics, Université de Mons, Mons, Belgium; ^11^ Cellular and Molecular Immunology, GIGA Institute, Université de Liège, Liege, Belgium

**Keywords:** bioluminescence, luciferase, coelenterazine, luciferin-binding protein, luminous system, Pennatulidae, Anthoptilidae, Funiculidae

## Abstract

Bioluminescence is the production of visible light by living organisms. It occurs through the oxidation of luciferin substrates catalysed by luciferase enzymes. Auxiliary proteins, such as fluorescent proteins and luciferin-binding proteins, can modify the light emitted wavelength or stabilize reactive luciferin molecules, respectively. Additionally, calcium ions are crucial for the luminescence across various species. Despite the large phylogenetic distribution of bioluminescent organisms, only a few systems have been comprehensively studied. Notably, cnidarian species of the *Renilla* genus utilize a coelenterazine-dependent luciferase, a calcium-dependent coelenterazine-binding protein and a green fluorescent protein. We investigated the bioluminescence of three sea pen species: *Pennatula phosphorea*, *Anthoptilum murrayi* and *Funiculina quadrangularis* (Pennatuloidea, Anthozoa). Their light-emission spectra reveal peaks at 510, 513 and 485 nm, respectively. A coelenterazine-based reaction was demonstrated in all three species. Using transcriptome analyses, we identified transcripts coding for luciferases, green fluorescent proteins and coelenterazine-binding proteins for *P. phosphorea* and *A. murrayi*. Immunodetection confirmed the expression of luciferase in *P. phosphorea* and *F. quadrangularis*. We also expressed recombinant luciferase of *A. murrayi*, confirming its activity. We highlighted the role of calcium ions in bioluminescence, possibly associated with the mechanism of substrate release at the level of coelenterazine-binding proteins. The study proposes a model for anthozoan bioluminescence, offering new avenues for future ecological and functional research on these luminous organisms.

**Figure:**
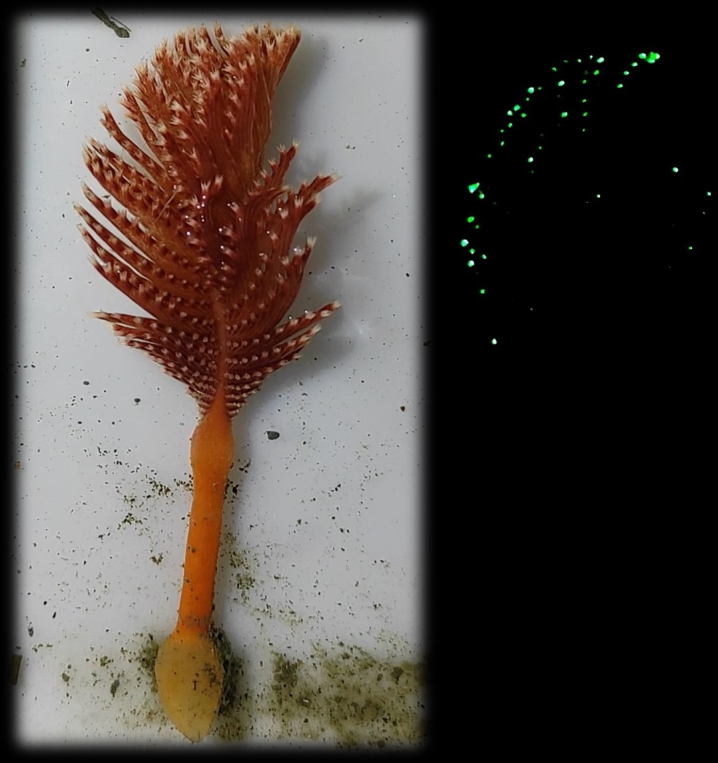

## 1. Introduction

Bioluminescence, the production of visible light by a living organism, involves the oxidation of a luciferin substrate catalysed by a luciferase (LUC) enzyme. In some bioluminescent systems, LUC and luciferin form a stable complex known as ‘photoprotein’, requiring additional co-factors to be functional [1]. LUCs *sensu lato* (i.e. LUCs *stricto sensu* and photoproteins) are classically considered taxon-specific (i.e. each clade is characterized by its own LUC) [2]. Nevertheless, homologous LUCs can sometimes be used by phylogenetically distant luminous organisms [3]. At least 12 distinct types of LUCs, *sensu lato*, have been described [3].

When the photogenic reaction occurs, the generated light is defined by a colour that can differ depending on luciferin, but also on the amino acid sequence and structure of the LUC [4–7]. In some bioluminescent organisms, light emission involves bioluminescence resonance energy transfer, a non-radiative process where a bioluminescent donor (LUC) transfers energy to a fluorescent acceptor (fluorescent protein) [4]. The fluorescent protein absorbs photons emitted by the bioluminescence reaction and becomes excited. It then re-emits light at longer wavelengths before returning to the ground state. For example, the green fluorescent protein (GFP) emits green light after absorbing blue light primarily emitted by the luciferin–LUC reaction [1,5].

Luminous anthozoans are distributed among four orders: Actiniaria (including one luminous family), Zoantharia (including two luminous families), Malacalcyonacea (including one luminous family) and Scleralcyonacea (electronic supplementary material, table S1) [8]. Scleralcyonacea include 18 luminous families mainly spread into the Pennatuloidea superfamily (electronic supplementary material, table S1) [8]. The bioluminescence system of *Renilla reniformis* (Pallas, 1766; Pennatuloidea) has been extensively characterized. It involves the most widespread luciferin in marine environments, the coelenterazine (CTZ) (6-(4-hydroxyphenyl)-2-[(4-hydroxyphenyl)methyl]-8-(phenylmethyl)-7*H*-imidazo [1,2-*a*]pyrazin-3-one), and a CTZ-dependent LUC (*R*Luc-type) [9–13]. This LUC is homologous to bacterial haloalkane dehalogenases, assumed to have been horizontally transferred during evolution [3]. In addition, more recent descriptions of similar bioluminescent molecular components in different pennatulaceans are sparse [9–17] (electronic supplementary material, table S1). Bessho-Uehara *et al*. [15] demonstrated the widespread occurrence of CTZ-based *R*Luc-type LUC across deep-sea pennatulacean species, such as *Distichoptilum gracile* (Verrill, 1882), *Umbellula* sp., *Pennatula* sp. and *Funiculina* sp. In addition, *R*Luc-type LUCs have also been identified in phylogenetically distant organisms, such as echinoderms (*Amphiura filiformis* (Müller, 1776) [18]), and potentially in tunicates (*Pyrosoma atlanticum* (Péron, 1804) [19]); however, this has recently been called into question [20]. Luminescence-associated molecules, such as CTZ-binding proteins (CBPs) and GFPs, have also been identified in sea pansies and other pennatulaceans [13,21–26]. Additionally, the calcium ion has been demonstrated to be indirectly involved in light production in *R. reniformis* and *Veretillum cynomorium* (Pallas, 1766) [27,28]. CBP relies on calcium ions as co-factors to release luciferin, enabling light production in *R. reniformis* [29].

Light production in sea pens occurs within specific endodermal cells (i.e. photocytes), depending on the species, in the tissues of autozooid and siphonozooid polyps [30–32]. These photocytes often exhibit green autofluorescence [33,34]. To date, bioluminescent pennatulaceans display nervous catecholaminergic control of light emission, with adrenaline- and noradrenaline-triggered waves of light flashes [35,36]. Although no experimental behavioural evidence has been obtained, the ecological function of pennatulacean luminescence may serve as an aposematic signal, avoiding predation through misdirection or burglar alarm effects [2,37]. The current data and gaps in the anthozoan bioluminescence data are presented in electronic supplementary material, table S1.

Although the *Renilla* bioluminescence system has been extensively characterized, the molecular mechanisms and components involved in the bioluminescence of other sea pen species remain largely unexplored. This study aimed to explore the unique bioluminescent systems of three sea pen species, the pennatulid *Pennatula phosphorea* (Linnaeus, 1758), funiculinid *Funiculina quadrangularis* (Pallas, 1766) and anthoptilid *Anthoptilum murrayi* (Kölliker, 1880), focusing on their biochemical and molecular aspects. A multidisciplinary study using luminometric assays, transcriptome and phylogenetic analyses, three-dimensional protein modelling and immunodetection revealed the basis of the bioluminescent system of these soft coral sea pens. Cross-reaction luminometric results highlight the involvement of an *R*Luc-like LUC in *P. phosphorea* and *F. quadrangularis*, both shallow water species, with CTZ as the substrate for all three bioluminescent systems. Besides, *P. phosphorea* and *A. murrayi de novo* transcriptome analyses corroborate (i) the homology of both retrieved LUC sequences with the *Renilla* LUC and (ii) the *in silico* presence of multiple CBPs-like and GFPs-like proteins homologous to the functionally characterized references proteins. The LUC expression sites within the autozooids and siphonozooids of *P. phosphorea* and autozooids of *F. quadrangularis* are described. The expression of GFP is also strongly suggested in *P. phosphorea* through comparative microscopy. Finally, the pharmacological involvement of calcium in the light-emission process of *P. phosphorea* and *F. quadrangularis* is confirmed.

## 2. Material and methods

### 2.1. Specimen collection

In July 2022 and 2023, common sea pens (*P. phosphorea*, *n* = 30) were collected from the Gullmarsfjord, Sweden, using a small dredge with a 1 m aperture at a depth of 35−40 m. Likewise, tall sea pens (*F. quadrangularis*, *n* = 11) were sampled in July 2023 using the same method at a depth of 40 m. Animals were transported to the Kristineberg Marine Research Station (Fiskebäckskil, Sweden) and maintained under dark conditions in a continuous flow of fresh deep-sea water pumped from the adjacent fjord.

Specimens of Murray’s sea pens (*A. murrayi*, *n* = 3) were collected during the DEEP-OCEAN expedition off the southeast coast of Brazil aboard the R/V Alpha Crucis. The collection was carried out using a demersal trawl net with 19 m lower rope, mesh sizes of 100 mm in the body and wings and 25 mm in the cod end, at an average depth of 1500 m. Collection permits were issued by the Instituto Chico Mendes de Conservação da Biodiversidade (SISBIO permit nos. 28054-4, 82624-1) and the Secretaria da Comissão Interministerial para Recursos do Mar da Marinha do Brasil (Portaria no. 223). After hoisting the net, all collected organisms were sorted, and *A. murrayi* specimens were promptly frozen in liquid nitrogen. These samples were stored at −80°C at the Oceanographic Institute of the University of São Paulo (São Paulo, Brazil).

### 2.2. Dissection and sample preparation

Specimens of *P. phosphorea* and *F. quadrangularis* were anaesthetized by immersion in 3.5% MgCl_2_ solution in sea water for 30 min [36]. For each tested specimen of *P. phosphorea*, (i) the pinnules were dissected and weighed and (ii) the rachis was divided into three equivalent portions and weighed. For specimens of *F. quadrangularis*, the rachis bearing the polyps was cut into 3 cm long segments. Sea pen pinnules and rachises were either used directly for biochemical assays or rinsed for 3 h in fresh, running deep-sea water for pharmacological calcium assays. Other specimens were fixed in 4% paraformaldehyde (PFA) in phosphate buffer saline (PBS; 123 mM NaCl, 2.6 mM potassium chloride (KCl), 12.6 mM Na_2_HPO_4_ and 1.7 mM KH_2_PO_4_ (pH 7.4)) for subsequent immunodetection techniques.

Due to the challenges of collecting organisms from great depths (e.g. trawling damage), maintaining the survival of *A. murrayi* was not feasible. Consequently, all measurements were conducted on frozen samples.

### 2.3. *In vivo* and *in vitro* light-emission spectrum

The bioluminescence spectrum of *A. murrayi* was obtained using frozen coral pieces thawed and hydrated with deionized water. The spectra were recorded using a cooled CCD camera (LumiF SpectroCapture AB-1850). Measurements were conducted in triplicate, with an exposure time of 2 min at room temperature.

Similarly, the emission spectrum of the recombinant *A. murrayi* LUC was recorded on the same equipment over a 60 s interval. Prior to this, the candidate *A. murrayi* LUC coding sequence (obtained based on §2.7) was optimized for *Escherichia coli* expression and cloned into the pET-28a vector (Biomatik) between the BamHI and HindIII restriction sites. The N-terminal His-tagged LUC was expressed in *E. coli* BL21 (DE3) cells, induced with 1  mM isopropyl-β-ᴅ-thiogalactopyranoside at 15°C overnight. Cells were harvested and lysed in 50  mM sodium phosphate buffer (pH 7.4) containing lysozyme. The lysate was then purified using Ni-NTA affinity chromatography with an imidazole gradient (100–500 mM). Final LUC preparations were pooled, dialysed against 50  mM sodium phosphate buffer (pH 7.4), screened for light-emission activity and concentrated using a 3 kDa cutoff filter (Vivaspin 500, GE Healthcare). Unless otherwise stated, protein concentrations were measured using a Qubit 3.0 fluorometer (Invitrogen). The luminescent reaction was then conducted under conditions identical to those of the standard light-emission assay. Specifically, 100  µl of solution containing *A. murrayi* LUC was diluted with 397  µl of 50  mM phosphate buffer (pH 7.4). The reaction was then initiated by adding 2  µl of native CTZ, bringing the total volume to 500  µl and yielding a final CTZ concentration of 6  µM.

### 2.4. Luminescent system biochemistry

Eleven specimens of *P. phosphorea* were used for biochemical analysis. Tests were performed for the pinnules (*n* = 40), rachises (*n* = 10) and peduncles (*n* = 10). Pinnule locations along the axis were observed and classified as upper, middle and lower, depending on the attachment position on the rachis, as in Duchatelet *et al*. [36]. Five specimens of *F. quadrangularis* were used immediately after dissection for biochemical assays. Measurements were performed independently of the polyp-bearing rachis location among the colonies (*n* = 10). Deep-frozen tissues from two *A. murrayi* specimens were used for this analysis. For the first two species, the central rigid calcified axis of the rachis was removed before starting experiments.

Light-emission measurements were performed in a dark room using an FB12 tube luminometer (Tirtertek-Berthold, Pforzheim, Germany) calibrated with a standard 470 nm light source (Beta light, Saunders Technology, Hayes, UK). Light responses were recorded using FB12-Sirius PC software (Tirtertek-Berthold). Light emission was characterized as follows: (i) maximum light intensity (*L*_max_), expressed in mega quanta per second (10^9^ q s^−1^), and (ii) total amount of light emitted (*L*_tot_) over 3 min, expressed in mega quanta. All data were standardized per unit mass (g).

### 2.5. Luciferase and coelenterazine assays

For the LUC assay, *P. phosphorea* pinnule and rachis and *F. quadrangularis* rachis were placed in 200 μl of Tris buffer (20 mM Tris and 0.5 mM NaCl; pH 7.4) and crushed with mortar and pestle until a homogenized extract was obtained; 20 and 40 μl of the extract were diluted in 180 and 160 μl Tris buffer, respectively. The diluted *P. phosphorea* and *F. quadrangularis* LUC solutions were injected into two different tubes filled with 5 μl of a 1/200 stock solution of CTZ (Prolume Ltd, Pinetop, AZ, USA) in cold methanol (1 optical density at 430 nm) diluted in 195 μl of Tris buffer. Two measurements of *L*_max_ were recorded and averaged to calculate the maximal light decay rate corresponding to LUC activity expressed in 10^9^ q s^−1^ g^−1^ [1].

For CTZ detection, *P. phosphorea* pinnules, rachises and *F. quadrangularis* rachises were placed in 200 μl of cold argon-saturated methanol and crushed using a mortar and pestle. Then, 5 μl of the methanolic extract was injected into a tube filled with 195 μl of Tris buffer and placed in a luminometer. Afterwards, 200 μl of *Renilla* LUC solution constituted of 4 μl of *Renilla* LUC (Prolume Ltd, working dilution of 0.2 g l^–1^ in a Tris–HCl buffer 10 mM, NaCl 0.5 M and bovine serum albumin (BSA) 1%; pH 7.4) and 196 μl of Tris buffer was injected into the luminometer tube. The *L*_tot_ was recorded and used to calculate the amount of CTZ contained in a gram of pinnule (ng g^−1^), assuming that 1 ng of pure CTZ coupled with *Renilla* LUC emits 2.52 × 10^11^ photons [1].

*Anthoptilum murrayi* tissues were processed using a Potter–Elvehjem homogenizer in 2 ml of Tris–HCl buffer (50 mM, pH 8.0). Following homogenization, the mixture was centrifuged at 15 000*g* for 10 min at 4°C, and the pellet was discarded. The supernatant was used for the light-emission assays. For each assay, the mixture comprised 100 µl of supernatant, 397 µl of Tris–HCl buffer (50 mM, pH 7.4) and 3 µl of CTZ (Prolume Ltd, Pinetop, AZ, USA), to achieve a final volume of 500 µl with a final concentration of 6 µM.

Control measurements were performed using CTZ in the presence of the buffer, and the emitted light remained around 1000 relative light units. This value was subtracted from the light emitted by the enzymatic assays for standardization purposes. All luminescence assays were conducted in triplicate, and the final result represents the average of the triplicates.

### 2.6. Long-term light monitoring of coelenterazine production

The *P. phosphorea* specimens (*n* = 12) were maintained in tanks filled with circulating artificial seawater (ASW; 400 mM NaCl, 9.6 mM KCl, 52.3 mM MgCl_2_, 9.9 mM CaCl_2_, 27.7 mM Na_2_SO_4_ and 20 mM Tris; pH 8.2) at temperatures following natural temperature variations encountered in the native fjord (https://www.weather.mi.gu.se/kristineberg/en/data.shtml) with a 12−12 h photoperiod. Sea pens were fed weekly with REEF LIFE Plancto (Aqua Medic, Germany), a food without any CTZ trace. After 6 and 12 months, KCl depolarization, CTZ content and LUC activity assays were performed on eight and four specimens, respectively, with two replicates per specimen. LUC and CTZ assays were performed as previously described. Total depolarization through KCl application allows for rapid estimation of the luminous ability of specimens. For the KCl experiments, luminescence induction was performed on the pinnule and rachis portions placed in a tube luminometer filled with 500 µl of ASW. Then, the light emission was triggered with the addition of 500 µl of a KCl solution (400 mM KCl, 52.3 mM MgCl_2_, 9.9 mM CaCl_2_, 27.7 mM Na_2_SO_4_ and 20 mM Tris; pH 8.2), and *L*_tot_ was recorded over 3 min. The recorded LUC activity, luciferin content and KCl response were compared with wild-caught measurements.

### 2.7. *De novo* transcriptome analyses

The pinnule, rachis and peduncle tissues of a single *P. phosphorea* specimen were dissected and directly immersed in a permeabilizing RNAlater-Ice (Life Technologies) solution overnight at −20°C, following the manufacturer’s protocol. Subsequently, the samples were stored at −80°C and processed for RNA extraction. Total RNA was extracted using the TRIzol reagent. The quality of the RNA extracts (RNA integrity number (RIN) value, fragment length distribution and 28S/18S ratio) and their concentrations were assessed using an Agilent 2100 bioanalyzer. The BGI company (Beijing Genomics Institute, Hong Kong) performed cDNA library preparation and sequencing using a procedure similar to that previously described [38–40]. High-throughput sequencing was conducted using the BGISEQ-500 platform to generate 100 bp paired-end reads. To exclude low-quality sequences, the raw reads were filtered by removing (i) reads with more than 20% of the qualities of the base lower than 10, (ii) reads only containing the adaptor sequence, and (iii) reads containing more than 5% of unknown nucleotide ‘N’. Quality control of the reads was performed using FastQC software [41]. For *P. phosphorea*, a reference *de novo* transcriptome assembly was then created from the remaining clean reads obtained from the pinnule, rachis and peduncle tissues using Trinity software [42] (Trinity-v2.5.1; min_contig_length 150, CPU 8, min_kmer_cov 3, min_glue 3, SS_lib_type RF, bfly_opts′-V 5, edge-thr = 0.1, stderr′). TGICL software was then used to reduce transcriptome redundancy by assembling the contigs into a single set of longer, non-redundant and more complete consensus unigenes [43] (Tgicl-v2.5.1; -l 40 c 10 v 25 -O′-repeat_stringency 0.95 -minmatch 35 -minscore 35′). Unigenes, defined as non-redundant assembled sequences obtained from assembly and/or clustering [44], can form clusters in which the similarity among overlapping sequences is greater than 70% or singletons that are unique unigenes. For all transcriptomes, unigene expression was evaluated using the ‘fragments per kilobase of the transcript, per million fragments sequenced’ (FPKM) method [38–40,44]. To obtain annotation for transcriptomes, unigenes were aligned to NCBI Nucleotide (NT), NCBI protein (NR), EuKariotic Orthologous Groups (KOG), Kyoto Encyclopedia of Genes and Genomes (KEGG) and UniProtKB/SwissProt databases using Blastn, Blastx [45] and Diamond [46]. Blast2GO [47] with NR annotation results was used to obtain Gene Ontology (GO) annotations according to molecular function, biological process and cellular component ontologies. InterPro [48] was also used to annotate unigenes based on functional analyses of protein sequences, clustering them into families and predicting the presence of domains or essential amino acid residues. The candidate coding area among the unigenes was assessed using Transdecoder (https://transdecoder.github.io).

A similar procedure was used for the specimen of *A. murrayi*. Whole sea pen tissues (pinnule and peduncle) were dissected, frozen in liquid nitrogen and stored at −80°C. The total RNA from the sea pen was extracted using the RNeasy Plant Mini Kit (QIAGEN), following the manufacturer’s instructions. RNA samples were treated with DNase I (Invitrogen) to remove potential genomic DNA contamination. Subsequently, to remove impurities and residual DNase I reaction remnants, they underwent a column cleanup process, following the instructions of the QIAGEN kit. The concentrations of RNA samples were estimated by fluorescence using a Qubit fluorometer (Thermo Fisher Scientific). The integrity of the RNA samples was confirmed by agarose gel electrophoresis (1%) stained with SYBR Safe (Invitrogen), and the RNA was dried at 30°C for 1 h in speed vac mode V-AQ.

The quality of RNA extracts (RIN value, fragment length distribution and 28S/18S ratio) and their concentration were assessed by RNA concentrations evaluated using an Agilent 2100 bioanalyzer. After QC, mRNA was enriched using oligo(dT) beads. First, mRNA was randomly fragmented by adding a fragmentation buffer. Then, the cDNA was synthesized using an mRNA template and random hexamer primer, after which a custom second-strand synthesis buffer (Illumina), dNTPs, RNase H and DNA polymerase I were added to initiate second-strand synthesis. After a series of terminal repairs, A ligation and sequencing adaptor ligation, the double-stranded cDNA library was completed through size selection and PCR enrichment. The GenOne Biotechnologies company (Brazil) performed cDNA library preparation and sequencing (150 bp paired-end reads). To exclude low-quality sequences, the raw reads were filtered by removing (i) reads with more than 50% of the qualities of the base lower than 5, (ii) reads only containing the adaptor sequence, and (iii) reads containing more than 10% of unknown nucleotide ‘N’.

RNA sequencing was performed with v4 2 × 100  bp reads on the HiSeq2500 platform by NGS Soluções Genômicas in Piracicaba, São Paulo. The reference *de novo* transcriptome assembly was generated using Trinity v2.13.2. To differentiate among identified isoforms, transcript abundance was analysed using the align_and_estimate_abundance.pl script from the Trinity software package [49].

Transcriptome completeness was evaluated using Benchmarking Universal Single-Copy Orthologs (BUSCO v4), a bioinformatics tool that determines the proportion of single-copy orthologues present in the dataset Metazoa_odb9. The BUSCO analysis and visualization of the results were conducted on the Galaxy platform (https://usegalaxy.org).

### 2.8. Bioluminescence-related protein sequence analyses

Potential transcripts of interest were chosen using NCBI online tools according to potential phylogenetic homologies to identify genes involved in the light production process, such as LUCs, GFPs and CBPs. These ‘light emission process-related genes’ were searched within the newly generated *P. phosphorea* transcriptome using tBLASTn analysis (1 hit, *E* value < 1 × 10^−20^). All retrieved unigenes were individually reciprocally searched in the NCBI NR database (Reciprocal BLASTx; 1hit, *E* value < 1 × 10^−20^). BLAST hits with significant *E* values strongly support homologous proteins. *In silico* translation (ExPASy translate tool, http://expasy.org/tools/dna.html) was performed on the sequences retrieved from the *P. phosphorea* and *A. murrayi* transcriptomes for all putative candidates. Multiple alignments were performed for each predicted protein with their respective homologous ‘light emission process-related protein’ retrieved from the NCBI online tool in other species using Geneious software [50]. Sequence alignments have enabled the identification of LUC characteristic features, such as catalytic triads [51].

To validate the *P. phosphorea* LUC retrieved sequence, primers were designed based on *Renilla muelleri* LUC mRNA (AY015988.1) to amplify the hypothetical LUC sequence using Primer BLAST software (electronic supplementary material, table S2). *Pennatula phosphorea* pinnules were collected and lysed in 300 µl of 50 mM NaOH for 30 min at 95°C with agitation at 800 r.p.m. The pH was adjusted by adding 60 µl of 500 mM Tris–HCl at pH 8. For PCR amplification, the reaction mix contained 1 U of Expand Long Template (Roche) with the provided buffer, 400 µM dNTP (R0191, Life Technologies) and 250 nM of each primer. Amplification was performed as follows: 95°C for 5 min; 35 cycles of denaturation at 95°C for 30 s, hybridization from 52°C to 55°C for 15 s and elongation at 68°C for 45−80 s. The final cycle was completed with a final elongation step at 68°C for 7 min. Primer pairs F1–R1, F2–R2 and F8–R2 resulted in amplifications visualized through electrophoresis and ethidium bromide incorporation. Genomic DNA was purified using the Qiagen PCR Purification Kit according to the manufacturer’s instructions and sequenced by Microsynth.

### 2.9. Phylogenetic analyses

The predicted sequences of the LUCs, GFPs and CBPs of *P. phosphorea* and *A. murrayi* were placed into an anthozoan-focused phylogenetic context using maximum likelihood phylogenetic reconstruction [52]. *Renilla*-type LUC sequences from selected metazoans were collected from public databases based on literature [15,17,18]. GFP sequences from cnidarians were collected from public databases using reference literature [53–56]. CBP sequences from anthozoans were collected from public databases based on literature [57]. Multiple alignments of all sequences were performed using the MAFFT algorithm implemented in Geneious software and trimmed using TrimAL software [58]. Maximum likelihood phylogenetic analyses were performed using IQ-tree software [59]. Before the analyses, ModelTest [60] was used to select the best-fit evolution model. Trees were edited using the iTOL Web tool.

### 2.10. Structural modelling

AlphaFold models of *P. phosphorea* and *A. murrayi* LUC, GFP and CBP proteins were obtained from the AlphaFold Web server (https://alphafoldserver.com/). The structural models were visualized using PyMOL 2.6 (https://pymol.org/). Structural pairwise alignments and calculations were performed using the Dali server (http://ekhidna2.biocenter.helsinki.fi/dali/).

### 2.11. Luciferase and green fluorescent protein immunodetection

Commercial antibodies against *Renilla* LUC (GTX125851, Genetex) were used to confirm the presence of a *Renilla*-like LUC. Proteins were extracted from frozen pinnules, rachis and peduncle samples. Each sample was homogenized on ice in 1000 μl of 2% Triton X-100 in PBS (10 mM Tris, pH 7.5; 1 mM EDTA, pH 8.0; 100 mM NaCl) supplemented with protease inhibitors (complete-Mini tablets; Roche). The extract was sonicated and centrifuged at 800*g* for 15 min. The supernatant was then collected. The protein concentration in each extract was measured using a Pierce™ BCA Protein Assay Kit (Thermo Fisher Scientific). Laemmli buffer (Bio-Rad) and β-mercaptoethanol (βMSH; Bio-Rad) were added to each protein extract and the proteins were electrophoretically separated at 200 V for 35 min on 12% sodium dodecylsulfate–polyacrylamide gel electrophoresis gels. The separated proteins were electroblotted onto nitrocellulose membranes. The membrane was incubated overnight with the primary anti-*Renilla* LUC antibody and the secondary antibody (enhance chemiluminescence horseradish peroxidase-conjugated anti-rabbit antibody; Life Sciences, NA934VS, lot number 4837492) for 1 h. Antibody detection was performed using the reagents of the detection kit (HRP Perkin-Elmer, NEL 104) following the manufacturer’s instructions. The dilution for the primary antibody was 1:2000.

The same primary antibody was used to immunolocalize LUCs within the *P. phosphorea* pinnules, rachis, peduncle tissues and *F. quadrangularis* polyp tissue. For whole-mount immunofluorescence, the dissected samples were blocked with PBS containing 2% Triton X-100 and 6% BSA (Amresco). Samples were then incubated for 48 h with the anti-*Renilla* LUC antibody diluted 1:200 in PBS containing 1% Triton X-100, 0.01% NaN_3_ and 6% BSA. Visualization of the LUC signal was performed after 24 h of incubation of the samples at room temperature in the dark with a fluorescent dye-labelled secondary antibody (Goat Anti-Rabbit, Alexa Fluor 594; Life Technologies Limited) diluted 1:500 in PBS containing 1% Triton X-100, 0.01% NaN_3_ and 6% BSA. Samples were mounted (Mowiol 4-88; Sigma) and examined using an epifluorescence microscope (Axio Observer; Zeiss, Oberkochen, Germany) equipped with Zen microscopy software (Zeiss, Oberkochen, Germany). Control sections were incubated in PBS containing 1% Triton X-100, 0.01% NaN_3_ and 6% BSA with no primary antibodies.

### 2.12. Calcium assays

Following the identification of the key proteins involved in the luminescence system, we assessed the role of calcium, another crucial element in the bioluminescence mechanism. Different pharmacological tests were used to investigate the role of calcium in the light-emission process of *P. phosphorea* and *F. quadrangularis* (*n* = 6 for each species). First, the calcium concentration was tested using three ASW solutions with different calcium concentrations (0, 10 and 20 mM CaCl_2_). To remove any traces of Ca^2+^ ions in the 0 mM CaCl_2_–ASW solution, calcium chelators EGTA (4100, Merck) and BAPTA (14513, Merck) were added to the solution at 10^–5^ M final concentrations. Second, the effect of a calcium ionophore (A23187; C7522, Merck) was tested to highlight the potential involvement of calcium storage in the light-emission process. Third, the involvement of calcium was tested in the presence of a previously determined triggering agent of light emission in sea pens, adrenaline at 10^–5^ M. Finally, calcium involvement was tested on the effect of the KCl depolarization solution usually employed to trigger the maximum light production in a bioluminescent species.

After rinsing for 3 h, the pinnules of *P. phosphorea* and polyp-bearing rachises of *F. quadrangularis* were placed in luminometer tubes and subjected to various treatments (electronic supplementary material, table S3). Before the experiments, each sample was pre-incubated for 15 min in ASW devoid of calcium (0 mM CaCl_2_). Data were recorded for 15 min using an FB12 tube luminometer (Tirtertek-Berthold, Pforzheim, Germany). Light emission was defined as the total amount of light emitted (*L*_tot_) over 15 min and was expressed in mega quanta. All data were standardized per unit mass (g).

### 2.13. Statistical analyses

All statistical analyses were performed with R Studio (v. 2023.03.1+446, 2022; Posit Software, USA). Variance normality and equality were tested using the Shapiro–Wilk test and Levene’s test, respectively. When these parametric assumptions were met, Student’s *t*‐test and ANOVA coupled with Tukey’s test were used to perform single or multiple comparisons between groups. When log transformation did not provide normality and homoscedasticity, the non-parametric Wilcoxon test and Kruskal–Wallis test coupled with the Wilcoxon rank-sum test were used to assess whether significant differences were present between the two groups or multiple groups. Differences were considered significant at a minimum *p*-value of < 0.05. Values are graphically illustrated as the mean and standard error of the mean.

## 3. Results

### 3.1. *Pennatula phosphorea*, *Funiculina quadrangularis* and *Anthoptilum murrayi* emit light using a coelenterazine-dependent luciferase

**Figure 1.**
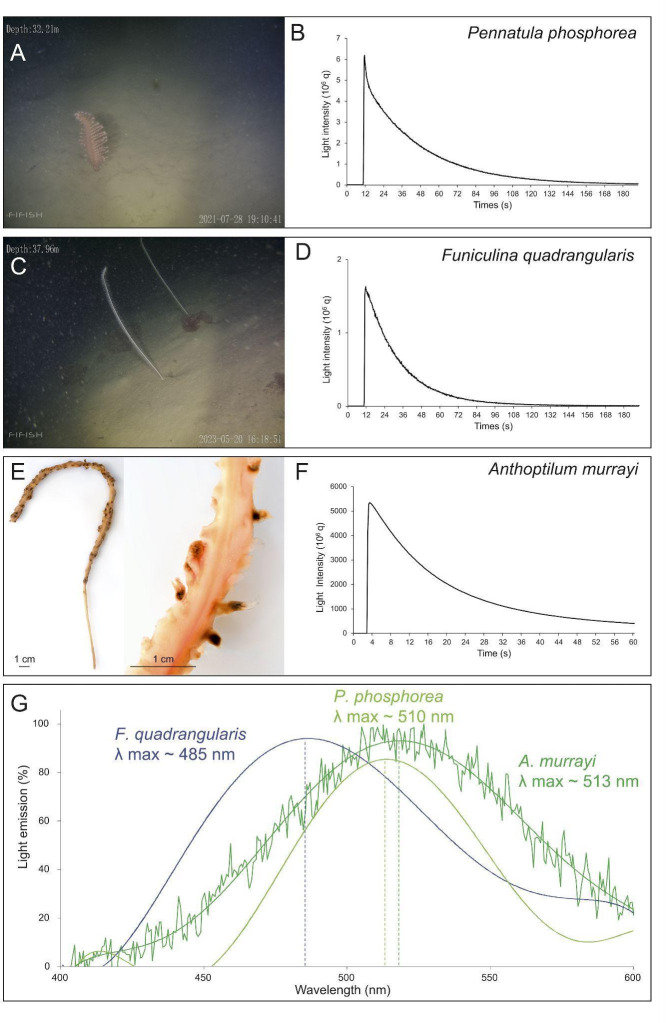
Biochemistry assays. (A) *In situ* images of *P. phosphorea* and (B) typical CTZ assay curve for the pinnules. (C) *In situ* images of *F. quadrangularis* and (D) typical CTZ assay curve for the 3 cm long polyp-bearing rachis. (E) Images of *A. murrayi* with a zoom on the polyp on the rachis and (F) typical CTZ assay curve for the whole specimen. (G) *Anthoptilum murrayi in vivo* luminescence spectrum compared with the retrieved spectra of two other species (*P. phosphorea* [61] and *F. quadrangularis* [15]). (A,C) Images provided by Fredrik Gröndahl.

Biochemical assays performed on *P. phosphorea*, *F. quadrangularis* and *A. murrayi* (figure 1A–F) demonstrated a similar response pattern. The cross-reactivity of the extracts with the commercial *Renilla* LUC highlights the involvement of CTZ in the bioluminescent systems of *P. phosphorea*, *F. quadrangularis* and *A. murrayi* (figure 1). For *P. phosphorea*, the measured amounts of CTZ present no statistical differences between the pinnule areas (ANOVA, *p*-value lower-middle = 0.8122; *p*-value lower-upper = 0.9054; *p-*value upper-middle = 0.7014; electronic supplementary material, figure S1). Similarly, no significant differences were observed between the rachis portions (ANOVA, *p*-value lower-middle = 0.5905; *p*-value lower-upper = 0.8274; *p*-value upper-middle = 0.4634; electronic supplementary material, figure S1). The mean CTZ contents per pinnule and rachis were 131.15 ± 29.06 and 70.00 ± 16.79 ng g^–1^, respectively. Peduncle presents an almost negligible mean value of 0.21 ± 0.01 ng g^–1^. Comparatively, the mean CTZ content of *F. quadrangularis* polyp-bearing rachis was 3.79 ± 1.20 ng g^–1^.

In parallel, a typical CTZ assay curve was observed for all three species (figure 1B,D,F). Cross-reaction assays using synthetic CTZ to measure potential LUC activity confirmed the involvement of a CTZ-based LUC system in *P. phosphorea* and *F. quadrangularis*. For *P. phosphorea*, LUC activities in the pinnules show no statistically significant differences across the pinnule areas (ANOVA, *p*-value lower-middle = 0.4963; *p*-value lower-upper = 0.2742; *p*-value upper-middle = 0.6251), with a mean *L*_max_ value of 129.5 ± 22.1 10^9^ q g^–1^ s^–1^ (electronic supplementary material, figure S1). Similarly, no statistically significant differences were observed in the *L*_max_ values recorded between rachis portions (ANOVA, *p*-value lower-middle = 0.6360; *p*-value lower-upper = 0.4438; *p*-value upper-middle = 0.2548), with a mean *L*_max_ value of 87.9 ± 16.9 10^9^ q g^–1^ s^–1^ (electronic supplementary material, figure S1). Finally, the peduncles tested exhibited a significantly lower mean *L*_max_ value (0.6 ± 0.4 10^9^ q g^–1^ s^–1^) compared to the rachis (ANOVA, *p* = 0.0298). Comparatively, *F. quadrangularis* LUC activity presents a mean *L*_max_ value of 313.8 ± 99.2 10^9^ q g^–1^ s^–1^. To complete the data on the sea pen light-emission spectrum, the *in vivo* light-emission spectrum of *A. murrayi* was measured and revealed a peak wavelength at 513 nm (figure 1G).

### 3.2. *Pennatula phosphorea* maintains its bioluminescence ability after 1 year in captivity without an exogenous supply of coelenterazine

Before the experiment, visual assessments of *P. phosphorea* luminescence were performed in the dark. These observations confirmed that the species maintained the ability to produce visible light even after 6−12 months of captivity without any external sources of CTZ-containing food.

Measurements of luminescence parameters showed a general decrease in all three parameters for both the pinnules and the rachis (electronic supplementary material, figure S2). For the pinnules, the maximum light emission shows statistically significant differences upon KCl application (Kruskal–Wallis test, *p* = 1.8 × 10^–5^), with a mean *L*_tot_ value of 361.7 ± 34.6 10^9^ q g^–1^ s^–1^ after field collection. This value is statistically different from the mean value observed after six months of captivity (Wilcoxon sum-rank test, *p* = 1.5 × 10^–6^) but not from the value after 12 months (Wilcoxon sum-rank test, *p* = 0.16). The mean *L*_tot_ values after 6 and 12 months are 80.5 ± 21.8 and 223.8 ± 84.4 10^9^ q g^–1^ s^–1^, respectively. CTZ content also presented statistically significant differences (Kruskal–Wallis test, *p* = 1.3 × 10^−5^). The mean CTZ content is already drastically reduced after 6 months (30.7 ± 4.5 ng g^–1^) but remains stable for 1 year (28.1 ± 4.4 ng g^–1^; Wilcoxon sum-rank test, T0 versus T6: *p* = 0.0001; T0 versus T12: *p* = 0.0004), without statistical differences between the former two (Wilcoxon sum-rank test, *p* = 0.91; electronic supplementary material, figure S2). Similarly, the LUC activities decreased over the year and presented statistically significant differences (Kruskal–Wallis test, *p* = 0.01). Differences occur between the initial *L*_max_ value (113.2 ± 12.7 10^9^ q g^–1^ s^–1^) and the mean *L*_max_ values of 77.0 ± 16.2 and 43.0 ± 3.7 10^9^ q g^–1^ s^–1^ after 6 and 12 months, respectively (Wilcoxon sum-rank test, T0 versus T6: *p* = 0.383; T0 versus T12: *p* = 0.013; T6 versus T12: *p* = 0.383; electronic supplementary material, figure S2). Similar observations occurred for the three parameters recorded on the rachis portions of *P. phosphorea* (electronic supplementary material, figure S2).

### 3.3. *De novo* transcriptomes of *Pennatula phosphorea* and *Anthoptilum murrayi*

For *P. phosphorea*, a total of 47.27 million raw reads of 200 bp length were generated from the pinnule library, 47.27 million from the rachis library and 41.96 million from the peduncle library. Data quality was assessed using FastQC software. Raw reads are available on the NCBI SRA database: *A. murrayi* (PRJNA1144931) and *P. phosphorea* (PRJNA1152785). After low-quality reads filtering, the remaining high-quality reads (i.e. 45.29 for the pinnule transcriptome, 45.53 for the rachis transcriptome and 40.29 for the peduncle transcriptome) were used to assemble a reference transcriptome using Trinity software. The obtained Trinity-predicted transcripts were clustered using TGICL to obtain the final unigenes.

**Figure 2.**
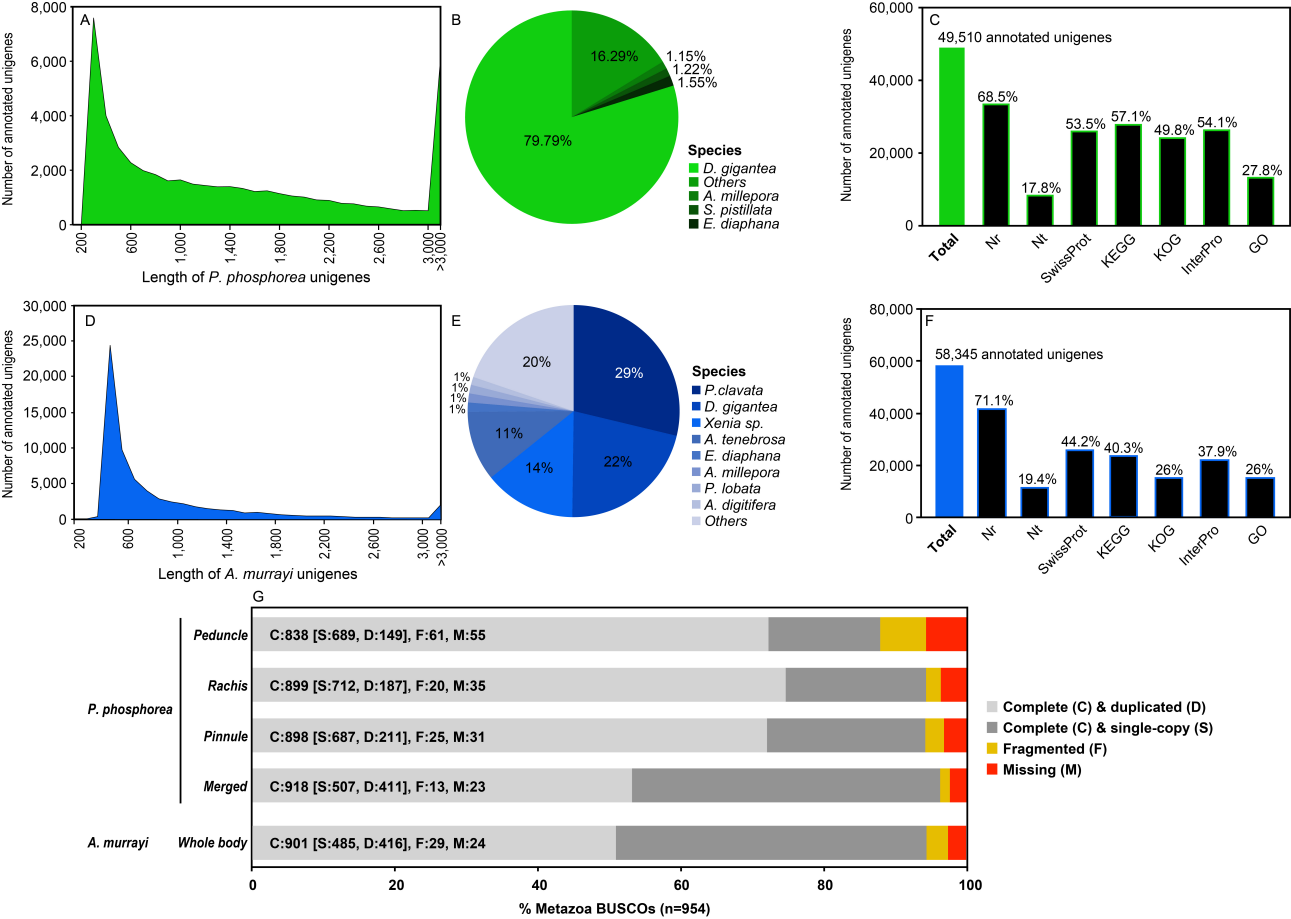
General description of the transcriptomic data. (A) The length distribution of *P. phosphorea* unigenes. (B) Taxonomic annotation of the *P. phosphorea* transcriptome. (C) Global annotation of *P. phosphorea* transcriptome. (D) Length distribution of the *A. murrayi* unigenes. (E) Taxonomic annotation of *A. murrayi* transcriptome. (F) Global annotation of the *A. murrayi* transcriptome. (G) BUSCO analyses.

In total, 49 510 unigenes (i.e. non-redundant unique sequences) were obtained with a total length of 72 928 901 bp. The average length was 1473 bp, and the N50 was 2350 bp. Among the transcriptome data, 35 439 unigenes for the pinnule dataset, 33 778 unigenes for the rachis dataset and 31 001 unigenes for the peduncle dataset were obtained, with a total of 49 510 different unigenes. The length distributions of the unigenes are shown in figure 2A, and the numerical data are summarized in electronic supplementary material, tables S4 and S5.

Of the 46 258 predicted unigenes, 1690 were found only in the pinnule transcriptome, 1114 only in the rachis transcriptome, 1035 in the peduncle transcriptome and 36 934 in the peduncle transcriptome. For descriptive purposes, comparative gene expression analysis was performed by mapping FPKM values (e.g. log_10_(FPKM value pinnule transcriptome) against log_10_(FPKM value rachis transcriptome)) calculated for all predicted unigenes (electronic supplementary material, table S6). However, it has to be clarified that transcriptome data have been generated for new gene discovery, not differential expression analyses, as no biological or technical replication was performed as a part of the study. The main species represented within the unigene annotation of the reference transcriptome was the anthozoan *Dendronephthya gigantea* (Verrill, 1864; 79%; figure 2B). Of the 49 510 *P. phosphorea* unigenes present in the filtered reference transcriptome, 34 984 showed significant matches with the molecular databases: 33 896 to NR (68.5%, *E* value < 1 × 10^–5^), 8818 to NT (17.8%), 26 473 to SwissProt (53.5%), 28 290 to KEGG (57.1%), 24 642 to KOG (49.8%), 26 790 to InterPro (54.1%) and 13 742 to GO (27.8%; figure 2C).

For *A. murrayi*, 66 425 unigenes were obtained, with a total length of 54 366 500 bp. The average mean length was 818 bp, and the N50 was 1140 bp. The length distributions of the unigenes are shown in figure 2D, and the numerical data are summarized in electronic supplementary material, tables S4 and S5. For descriptive purposes, gene expression analysis was performed by mapping the FPKM values (e.g. log_10_(FPKM value)) calculated for all predicted unigenes (electronic supplementary material, table S6). The main represented species within the unigene annotation of the reference transcriptome was the anthozoan *Paramuricea clavata* (29%; figure 2E). Among the 66 425 *A. murrayi* unigenes present in the filtered reference transcriptome, 58 345 were significantly matched to the molecular databases: 41 512 to NR (71.15%, *E* value < 1 × 10^−10^), 11 345 to NT (19.44%), 25 769 to SwissProt (44.16%), 23 549 to KEGG (40.36%), 15 178 to KOG (26.01%), 22 134 to InterPro (37.93%) and 15 178 to GO (26.01%; figure 2F).

Because of the lack of a reference genome in *P. phosphorea* and *A. murrayi* and possibly the relatively short length of some unigene sequences, 29.4% and 71.6%, respectively, of the assembled sequences could not be matched to any known genes.

Based on BUSCO analysis, 96.23% of metazoa BUSCO genes were predicted to be complete in the merged *P. phosphorea* transcriptome. In parallel, 1.36% of BUSCO genes were fragmented and 2.41% were missing (figure 2G). Similar results were obtained for *A. murrayi*, with 94.44% complete BUSCO genes found, while 3.04% of BUSCO genes were fragmented and 2.52% were missing (figure 2G).

**Figure 3.**
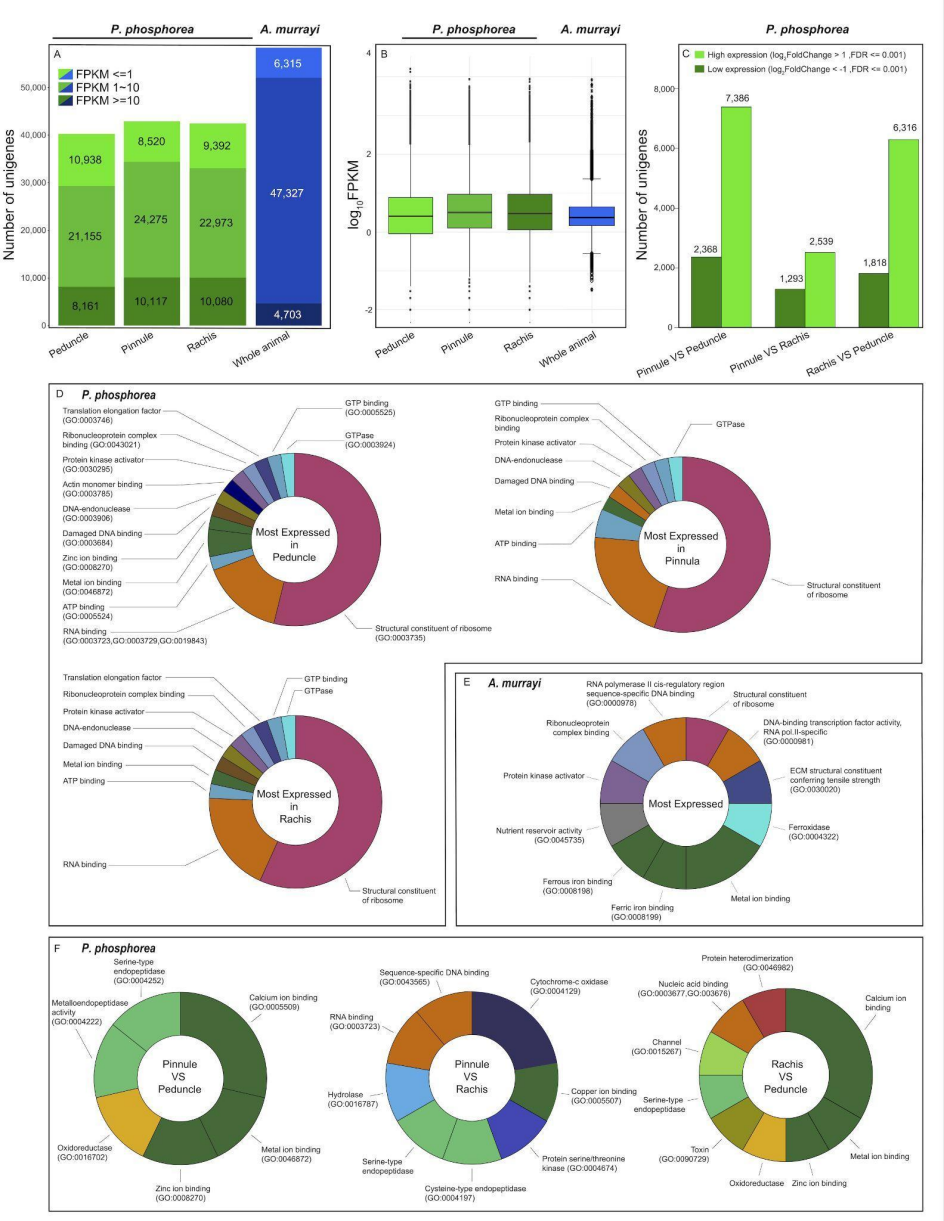
Global gene expression and Gene Ontology of *P. phosphorea* and *A. murrayi* transcriptomic data. (A) Distribution of FPKM expression values across the *P. phosphorea* and *A. murrayi* transcriptomes. (B) Gene expression distribution for each sample. (C) Proportions of unigenes with relatively high or low expression levels in *P. phosphorea* samples. Gene Ontology distribution of the 40 most highly expressed unigenes for each sample of *P. phosphorea* (D) and *A. murrayi* (E). (F) Comparison of Gene Ontology repartition of the 40 most highly expressed unigenes in *P. phosphorea* samples.

Analyses of unigene expression revealed a similar proportion of FPKM values in the three tissues (figure 3A,B). Comparatively, a large number of unigenes appeared to be more highly expressed in the pinnule and rachis than in the peduncle tissue (figure 3C). The pinnule and rachis tissues appeared to be more similar in terms of the unigene expression profile. The ‘Molecular function’ GO functional annotations for the 40 most expressed unigenes of each sample of *P. phosphorea* (figure 3D) and the whole-animal sample for *A. murrayi* (figure 3F) show a classical high expression of molecular actors involved in the cellular machinery and gene regulation. The GO functional annotations for the 40 unigenes with the most pronounced expression differences between tissues, including those highly expressed in one tissue relative to the other and those with lower expression levels, are shown in figure 3F. We did not perform formal differential gene expression analysis, as no replication was performed. Calcium ion-binding function (GO: 0005509) appeared to be predominantly expressed in pinnule and rachis tissues, compared to the peduncle (figure 3F). Similarly, the pinnule tissue (containing the feeding polyp) presented a higher proportion of genes annotated as digestive enzymes (figure 3F).

### 3.4. Expression of bioluminescence-related genes in *Pennatula phosphorea* and *Anthoptilum murrayi*

The *P. phosphorea* and *A. murrayi* transcriptomes contained sequences of several predicted LUCs, GFPs and luciferin-binding proteins. The FPKM values retrieved from each transcriptome are shown in electronic supplementary material, table S6. Reciprocal BLAST analyses revealed that the sequences matched the LUCs, CBPs and GFPs of anthozoans.

**Figure 4.**
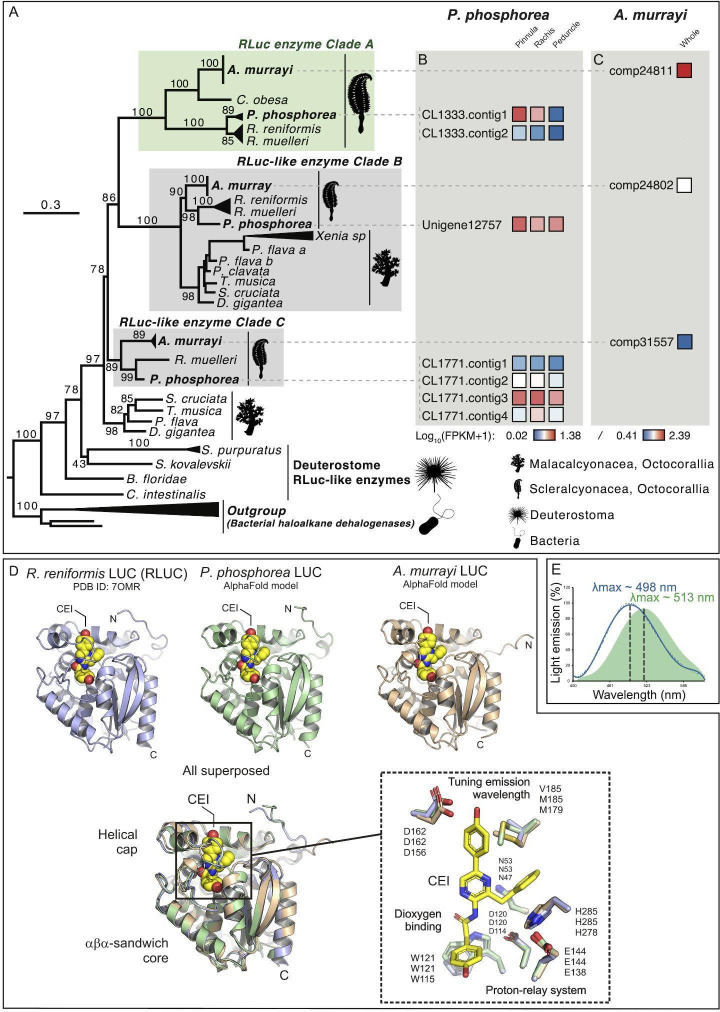
Phylogenetic tree of *R*Luc-like enzymes, including *P. phosphorea* and *A. murrayi* amino acid sequences. (A) Maximum likelihood tree based on the amino acid sequence alignment of *R*Luc-like enzymes. The tree was calculated by IQ-tree software using the LG+I+G4 model of evolution. Numbers at the nodes indicate ultrafast bootstrap values based on 1000 replicates. The scale bar represents the percentage of amino acid substitutions per site. Bacterial haloalkane dehalogenase sequences were used to root the tree. (B) The expression level of each retrieved *R*Luc-like sequence in a distinct portion of *P. phosphorea*. (C) Expression level of each retrieved *R*Luc-like sequence in the whole *A. murrayi* specimen. (D) Structural comparison of AlphaFold models of *P. phosphorea* and *A. murrayi* Luc proteins and the crystal structure of *R. reniformis* Luc complexes with coelenteramide (CEI) oxyluciferin (PDB ID: 7OMR). (E) Superimposed spectrum of the *in vivo* luminescence of *A. murrayi* (green) and the *in vitro* luminescent assay of *A. murrayi* LUC in the presence of CTZ (fc.: 6 µM; blue).

Several *R*Luc-like enzymes were recovered from both investigated species (*A. murrayi* and *P. phosphorea*). In addition, sequence mining allowed us to recover additional *R*Luc-like sequences from the genomes of *R. muelleri* and *R. reniformis*. Phylogenetic analyses revealed three clades of *R*Luc-like enzymes in Pennatulacea (figure 4A). Clade A contains well-known LUCs from *R. muelleri* and *R. reniformis* and probable LUC sequences from *A. murrayi*, *P. phosphorea* and *Cavernularia obesa* (Valenciennes, 1850). Interestingly, clade A did not contain any sequences from the non-luminous species. Clade B, in comparison, includes several sequences from non-luminous species (e.g. *Pinnigorgia flava* (Nutting, 1910) and *Sinularia cruciata* (Tixier-Durivault, 1970)), in addition to several sequences from both our model species. Clade C also contained the *R*Luc-like enzymes retrieved from each transcriptome.

The retrieved sequence (CL1333; figure 4B) of *P. phosphorea* LUC has an estimated molecular weight of 35.84 kDa. A comparison of the amino acid sequences of *P. phosphorea* and anthozoan LUCs demonstrated the presence of the catalytic triad involved in LUC activity (electronic supplementary material, figure S3 and file S1). These key sites consist of an aspartate residue in position 120, a glutamate residue in position 144 and a histidine in position 285. Residues asparagine 53 and tryptophan 121 (N53 and W121), previously established for the O_2_ stabilization and the CO_2_ release within *Renilla* LUC [12], were also retrieved in our LUC sequences (electronic supplementary material, figure S3). The retrieved *P. phosphorea* LUC sequence, based on RNA-seq data, appears to be highly similar to other known anthozoan LUCs. It shares 90.26% identity and 95% similarity with *R. reniformis* LUC (*Renilla*-luciferin 2-monooxygenase). This sequence was validated by DNA amplification and sequencing using *R*Luc primers. FPKM analyses revealed that this sequence was mostly expressed in the pinnule and rachis (figure 4B).

The *A. murrayi* LUC (Comp24811; figure 4C), with a molecular weight estimated at 34.61 kDa and consisting of 304 amino acids, exhibits 58% sequence identity and 98% coverage compared to *R*Luc, highlighting significant similarities. Sequence alignment of *A. murrayi* LUC with *R*Luc has revealed the conservation of the catalytic triad and active site. The *R*Luc structure features two distinct domains: a cap domain and alpha/beta-hydrolase domain. Key residues essential for enzymatic activity, including the substrate entry tunnel and catalytic triad (D120, E144 and H285), are located in the cap domain (electronic supplementary material, figure S3). These elements have also been identified in the LUC sequence of *A. murrayi*, as reported by Rahnama *et al*. [62] and Khoshnevisan *et al.* [63] for *Renilla* LUC. Through the expression of recombinant LUC in *E. coli* using degenerate primers derived from *A. murrayi* transcriptome analysis, *A. murrayi* LUC was tested for preliminary downstream expression, yielding an active enzyme capable of producing blue light (*λ*_max_ = 498 nm) upon CTZ addition (figure 4E).

Structural models of *P. phosphorea* and *A. murrayi* LUCs show a canonical aba-sandwich fold with a helical cap domain (figure 4D). Overall comparison between the crystal structure of *R. reniformis* LUC complexed with coelenteramide (CEI) oxyluciferin and structural models of *P. phosphorea* and *A. murrayi* LUCs showed root-mean-square deviation (RMSD) on the C_a_-atoms of 0.7 and 1.3, respectively. Careful inspection of the modelled LUC structures revealed that key residues of the catalytic pentad are conserved and properly positioned for productive catalysis (figure 4D). From these, three residues (aspartate–histidine–glutamate) function as a protein-relay system protonating a CEI oxyluciferin at amide nitrogen, while the two residues (asparagine and tryptophan) are responsible for co-substrate (dioxygen) binding. Moreover, an aspartate residue, responsible for tuning the emission wavelength, found on the rim of the catalytic pocket in *R. reniformis* LUC [12], is also conserved in these luminescent species.

**Figure 5.**
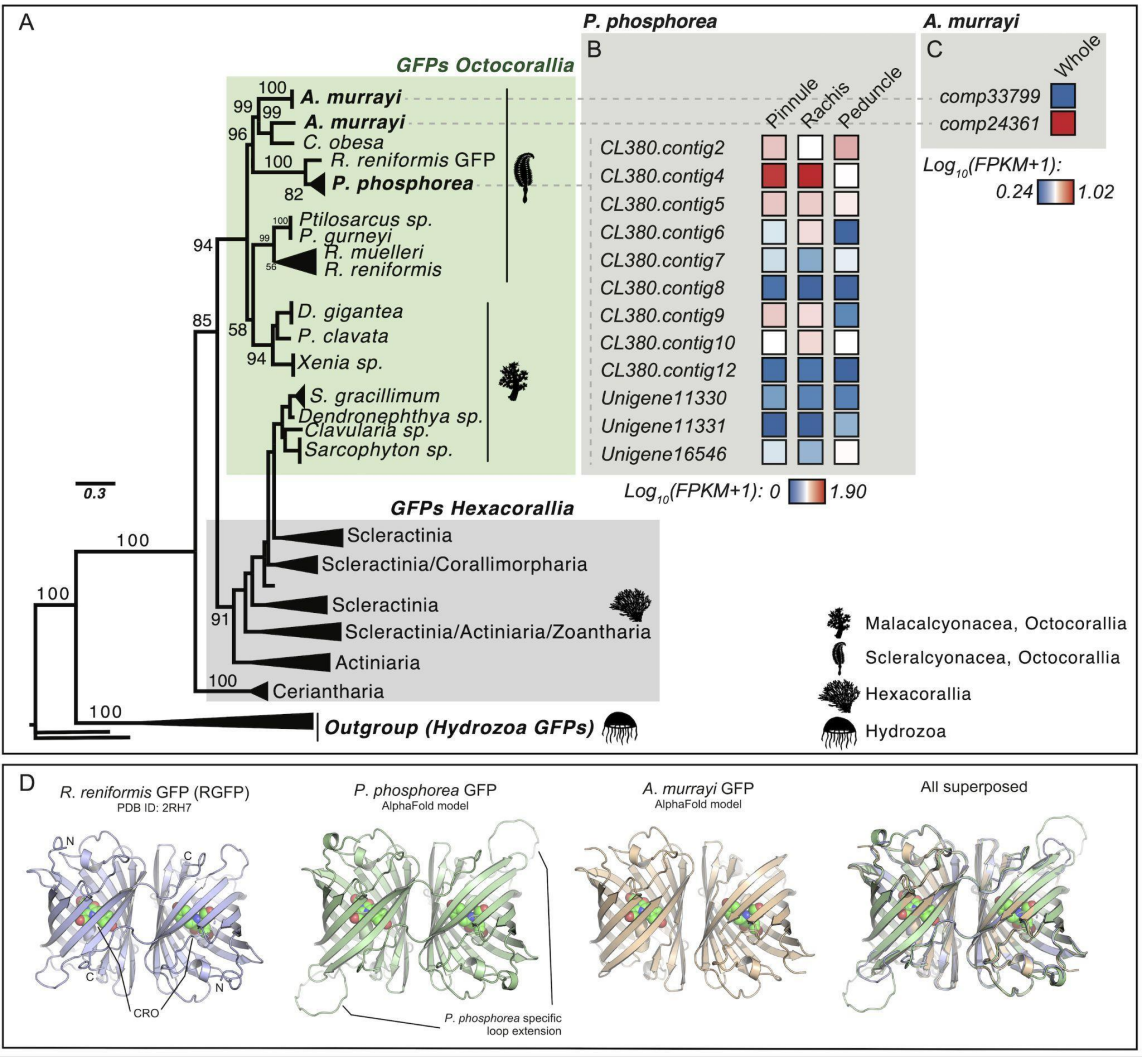
Phylogenetic tree of anthozoan fluorescent proteins, including *P. phosphorea* and *A. murrayi* amino acid sequences. (A) Maximum likelihood tree based on GFP amino acid sequence alignment. The tree was calculated by IQ-tree software using the WAG + R4 model of evolution. Numbers at the nodes indicate ultrafast bootstrap percentages based on 1000 replicates. The scale bar represents the percentage of amino acid substitutions per site. Hydrozoan GFP sequences were used to root the tree. (B) The expression level of each retrieved GFP sequence for a distinct portion of *P. phosphorea*. (C) The expression level of each retrieved GFP sequence in the whole *A. murrayi* specimen. (D) Structural comparison of AlphaFold models of *P. phosphorea* and *A. murrayi* GFP proteins and the crystal structure of *R. reniformis* GFP (PDB ID: 2HR7).

Several sequences coding for GFP-like sequences were retrieved from *the P. phosphorea* and *A. murrayi* transcriptomes. *Pennatula phosphorea* GFP (CL380; 20.81 kDa) and *A. murrayi* GFP (Comp24361; 27.24 kDa) appear homologous to other anthozoan sequences, and both sequences clustered with GFP sequences of bioluminescent Scleralcyonacea (figure 5A; electronic supplementary material, figure S3 and file S2). *Pennatula phosphorea* GFP shares 79.42% identity and 86% similarity with the GFP of *R. reniformis*. In comparison, *A. murrayi* GFP shared 73.06% identity and 87% similarity with the GFP of *C. obesa*. Pinnules and rachises were the main sites of *P. phosphorea* GFP expression (figure 5B). Interestingly, a perfect sequence repetition was observed in the most expressed *P. phosphorea* GFP sequence (electronic supplementary material, figure S3). Among the unigene pairs found in the *A. murrayi* transcriptome, Comp24361 appeared to be more highly expressed (figure 5C). Sequence analysis pinpointed a lack of N-terminal part for the *Anthoptilum* sequence (electronic supplementary material, figure S3).

Structural models of *P. phosphorea* and *A. murrayi* GFPs show a characteristic β-barrel fold with a fluorophore moiety ( peptide derived chromophore) covalently bound inside the barrel (figure 5D). *In silico* modelling suggests that these proteins may associate as homodimers, similar to the homodimeric structure observed in the crystal structure of *R. reniformis* GFP. However, further experimental validation is required to confirm this hypothesis. Comparison between the crystal structure of *R. reniformis* GFP and models of *P. phosphorea* and *A. murrayi* GFPs showed RMSD on the C_a_-atoms of 0.9 and 1.1, respectively, highlighting their high similarities. A structural feature distinguishing *P. phosphorea* and *A. murrayi* GFPs from their *R. reniformis* counterpart is the composition of the fluorophore moiety. While *R. reniformis* fluorophore is generated from a serine–tyrosine–glycine tripeptide, the fluorophores of *P. phosphorea* and *A. murrayi* are formed from a glutamine–tyrosine–glycine tripeptide, which may affect the fluorescent properties of these proteins. Moreover, there is one markedly extended solvent-exposed loop in *P. phosphorea* GFP (figure 5D). We believe that this species-specific extension might affect protein–protein complexation during the radiationless resonance energy transfer process, but future experimental evidence is needed to verify this hypothesis.

**Figure 6.**
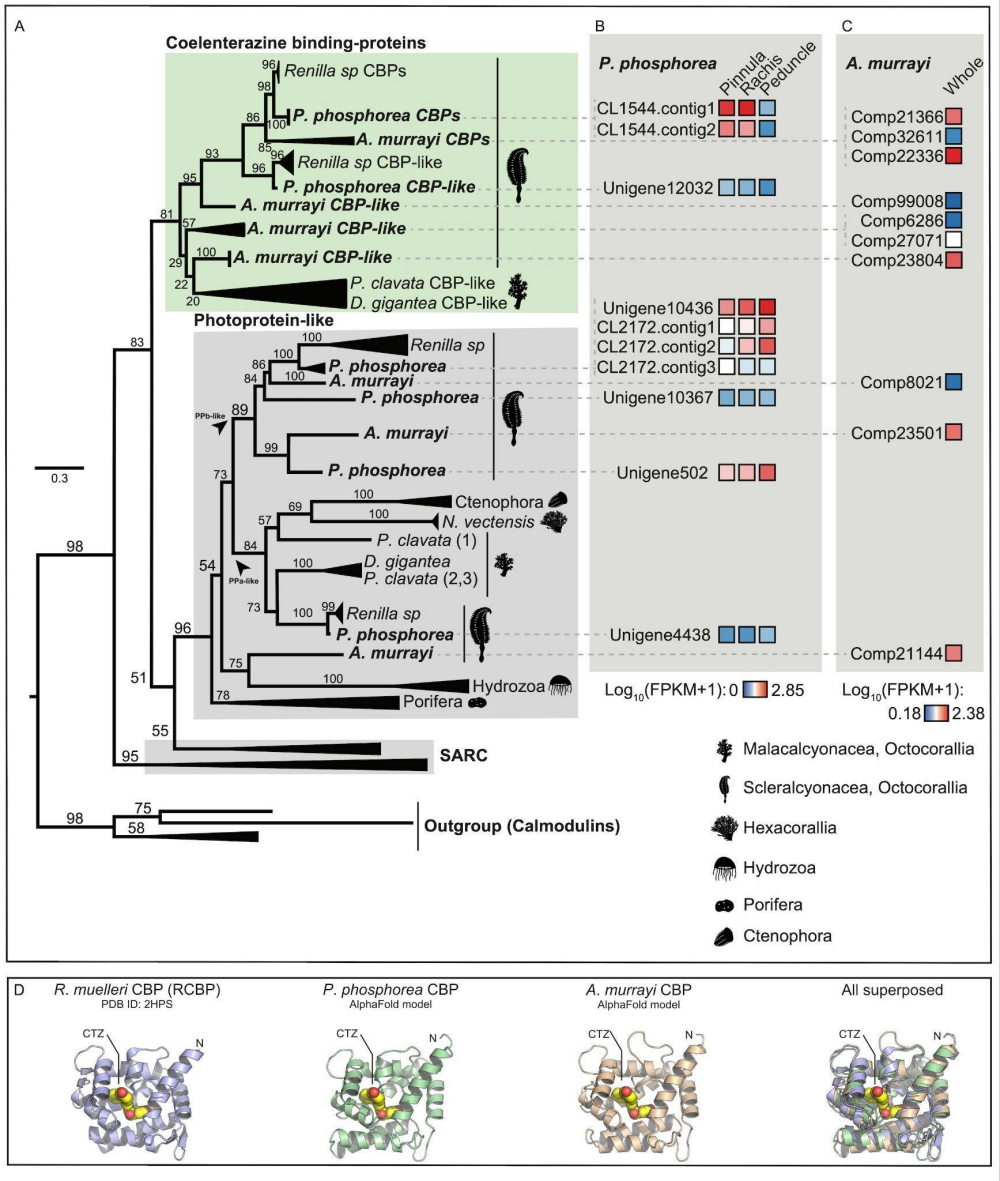
Phylogenetic tree of CBPs, including *P. phosphorea* and *A. murrayi* amino acid sequences. (A) Maximum likelihood tree based on the amino acid sequence alignment of CBPs. The tree was calculated by IQ-tree software using the LG + R4 model of evolution. Numbers at the nodes indicate ultrafast bootstrap percentages based on 1000 replicates. The scale bar represents the percentage of amino acid substitutions per site. Calmodulin sequences were used to root the tree. (B) The expression level of each retrieved CBP and CBP-like sequence for a distinct portion of *P. phosphorea*. (C) Expression level of each retrieved CBP and CBP-like sequence in the whole *A. murrayi* specimen. (D) Structural comparison of AlphaFold models of *P. phosphorea* and *A. murrayi* CBP proteins and the crystal structure of *R. muelleri* CBP complexes with CTZ luciferin (PDB ID: 2HPS).

Different CBP and CBP-like sequences were retrieved from the transcriptomes of *P. phosphorea and A. murrayi*. Some of these sequences clustered with the CBPs of luminous Scleralcyonacea, whereas others were found to be clustered with photoprotein-like proteins (figure 6A and electronic supplementary material, file S3). One sequence appeared to be mainly expressed within the *P. phosphorea* pinnule and rachis (CL1544; 26.84 kDa) and *the A. murrayi* colony (Comp 22336; 21.08 kDa; figure 6B,C). The most highly expressed *P. phosphorea* CBP appeared highly similar to other luminous anthozoan sequences. *Pennatula phosphorea* CBP shared 85.33% identity and 94% similarity with the luciferin-binding protein of *R. reniformis*, while *A. murrayi* CBP shared 50% identity and 74% similarity with the luciferin-binding of *R. reniformis*. Interestingly, sequences clustered in the photoprotein-like protein group appeared to be mainly expressed within the non-photogenic peduncle tissue in *P. phosphorea*.

Finally, structural models of *P. phosphorea* and *A. murrayi* Ca^2+^-regulated CBPs reveal a typical EF-hand fold, containing three Ca^2+^-binding sites. As shown in figure 6D, comparison between the crystal structure of *R. reniformis* CBP complexed with CTZ luciferin and structural models of *P. phosphorea* and *A. murrayi* LUCs show a structural similarity, with RMSD on the C_a_-atoms of 2.3 and 3.2, respectively. The RMSD values were higher than those observed for LUC and GFP proteins, but this is likely caused by a large conformational space that is searched by CBP proteins. Importantly, our modelling reveals an internal cavity in *P. phosphorea* and *A. murrayi* CBPs that is capable of accommodating CTZ luciferin, suggesting that these proteins are indeed functional luciferin-binding proteins (figure 6D).

### 3.5. Luciferase expression and green autofluorescence in *Pennatula phosphorea* and *Funiculina quadrangularis*

Based on the high sequence similarity of *P. phosphorea* LUC with *Renilla* LUC, a commercial anti-*Renilla* LUC antibody was selected for immunodetection. Immunoblot analyses revealed strong anti-LUC immunoreactive bands in both extracts of the pinnule and rachis tissues (electronic supplementary material, figure S4). The bands correspond to a protein with an approximate molecular weight of 35 kDa, matching the molecular weight of the predicted *P. phosphorea* LUC and the *Renilla* LUC molecular weight of 36 kDa. No labelling was detected in the peduncle tissue extract (electronic supplementary material, figure S4).

**Figure 7.**
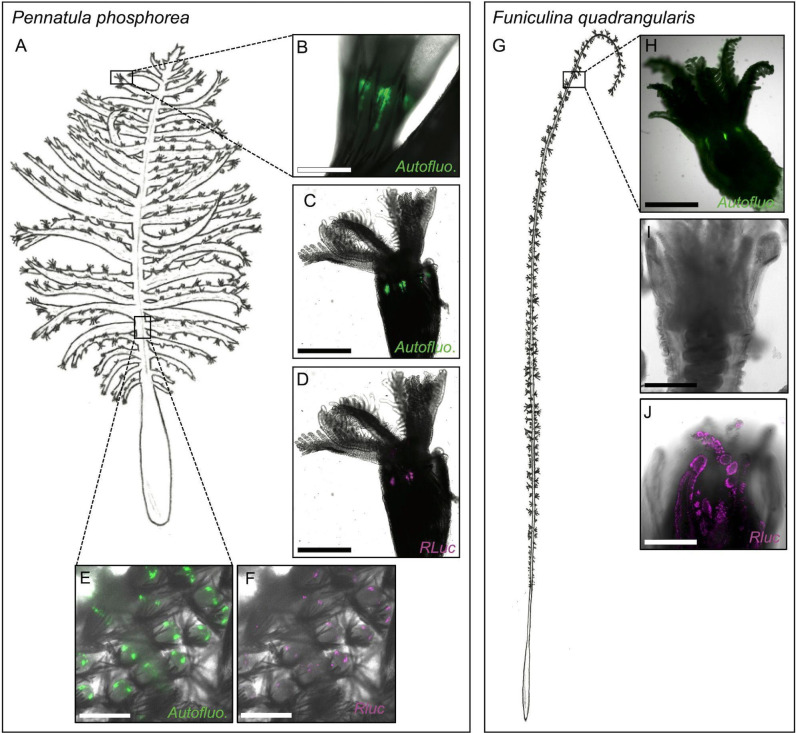
Autofluorescence and LUC immunodetection in *P. phosphorea* and *F. quadrangularis.* (A) Schematic illustration of *P. phosphorea*. Natural green autofluorescence (B), green fluorescence after fixation (C) and LUC immunodetection (D; magenta) of the *P. phosphorea* pinnule autozooids. Green fluorescence after fixation (E) and LUC immunodetection (F; magenta) in *P. phosphorea* rachis siphonozooids. (G) Schematic illustration of *F. quadrangularis*. Natural green autofluorescence (H), observation after fixation with no autofluorescence signal (I) and LUC immunodetection (J; magenta) of the *F. quadrangularis* autozooids. Scale bars, (B–F,H) 500 μm and (I,J) 250 μm.

On autozooid polyps of the pinnules (figure 7A–D), a strong green autofluorescence signal was observed at the tentacle crown base before (figure 7B) and after (figure 7C) PFA fixation. This fluorescent signal is located in clusters of cells at the tentacle junctions. Strong anti-*Renilla* LUC immunoreactivity was observed at the same level as the green fluorescence signal (figure 7C,D). On siphonozooid polyps of the rachis, the autofluorescence signal was also observable and was located as green dots (from 10 to 25 µm diameter) spread in the tissue, generally in pairs (figure 7E). Anti-*Renilla* LUC-positive cells colocalized with these autofluorescent dots (figure 7E,F). Finally, no green fluorescence or immunolabelling was detected in peduncle tissue (data not shown).

For *Funiculina* polyps (figure 7G), a green autofluorescence signal was observed in the freshly dissected specimens before fixation (figure 7H). This green autofluorescence completely disappeared after fixation (figure 7I). LUC localization differed from that in *P. phosphorea*. A strong LUC signal was detected within the polyp tentacle tissue and not at the base of the polyp crown (figure 7I,J).

Negative controls with the omission of the primary antibody did not reveal any non-specific binding of the secondary antibodies (data not shown).

### 3.6. Calcium is involved in the bioluminescence of *Pennatula phosphorea* and *Funiculina quadrangularis*

**Figure 8.**
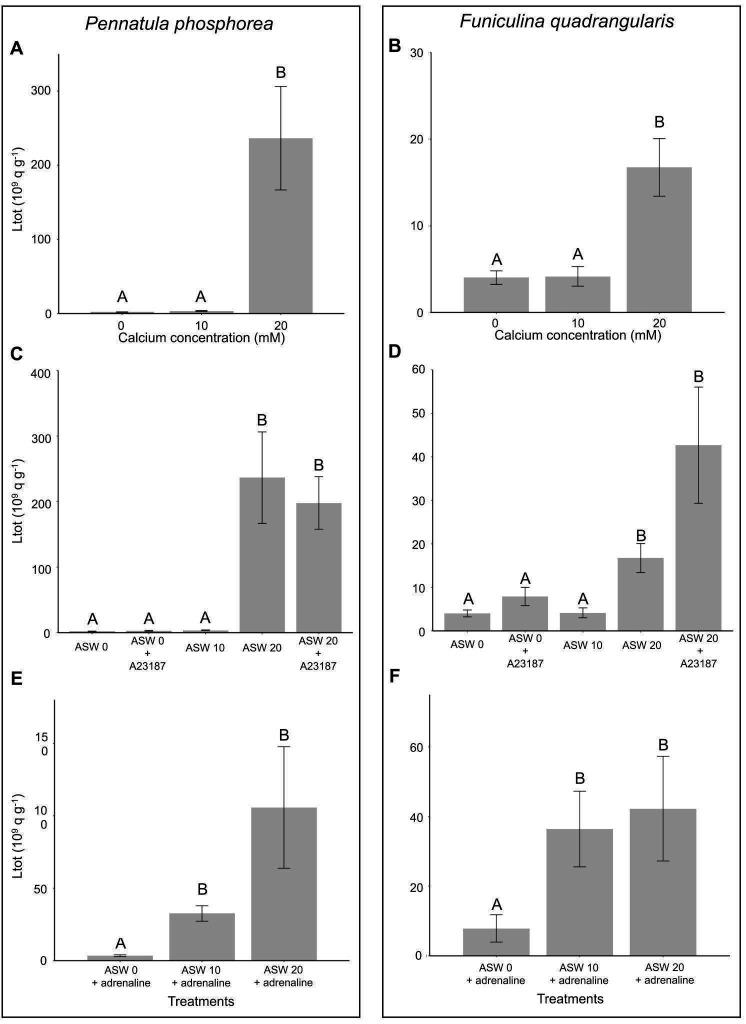
Calcium involvement in *P. phosphorea* and *F. quadrangularis* light emissions. Experiments were performed on *P. phosphorea* (A,C,E) and *F. quadrangularis* (B,D,F). Effect of different concentrations of calcium (0, 10 and 20 mM) in the medium on the total light emission (*L*_tot_) (A,B). Effect of different calcium concentrations (0, 10 and 20 mM) in the medium in the presence and absence of the calcium ionophore A23187 on the *L*_tot_ (C,D). Effect of different concentrations of calcium (0, 10 and 20 mM) in the medium in the presence of adrenaline (10^−5^ mol l^−1^) on the *L*_tot_ (E,F). Different lettering indicates statistical differences.

Based on *P. phosphorea* CBP retrieval in the *Pennatula* transcriptome data and the literature mentioning the potential implication of Ca^2+^ in the release of luciferin from CBPs, Ca^2+^ involvement in the light-emission process of *P. phosphorea* and *F. quadrangularis* was investigated. The tested specimens of both species revealed a drastic increase in light production when immersed in ASW with a doubled Ca^2+^ ion concentration (20 mM; figure 8A,B). At the same time, no statistical differences were observed between the normal ASW (10 mM) and ASW devoted to Ca^2+^ ions (0 mM; figure 8A,B). Analysis of the effects of the A23187 ionophore showed no statistical differences compared to the calcium concentration (figure 8C,D). For both specimens, adrenaline triggered light production when calcium was present in ASW (figure 8E,F). While an increase in the mean *L*_tot_ was observed between adrenaline-tested samples at 10 and 20 mM of CaCl_2_ in the medium, this increase was not statistically supported (figure 8E,F). Finally, no significant differences were observed in *L*_tot_ after KCl application at the three Ca^2+^ concentrations (electronic supplementary material, figure S5).

## 4. Discussion

Through biochemical cross-reaction experiments, this study demonstrated that the bioluminescence of *P. phosphorea*, *F. quadrangularis* and *A. murrayi* is associated with a CTZ-dependent LUC homologous to the well-known LUC of the sea pansy *Renilla*. The measured concentration of CTZ in *P. phosphorea* pinnules closely aligns with the reported values of 141 and 282.4 ng g^–1^ for the phylogenetically closely related species *R. muelleri* and *C. obesa*, respectively [1]. Similarly, the mean LUC activity of *P. phosphorea* pinnules reaches a comparable range to that of other organisms using *Renilla*-type LUCs such as *R. muelleri* (± 130 10^9^ q g^–1^ s^–1^) and *C. obesa* (± 220 10^9^ q g^–1^ s^–1^), or the sympatric ophiuroid *A. filiformis* (± 69 10^9^ q g^–1^ s^–1^) [1,64]. While the LUC activity recorded for *F. quadrangularis* is higher than for *P. phosphorea*, this species exhibits a smaller CTZ content, being closer to recorded values of the echinoderms *A. filiformis* (5.4 ng g^–1^ [64]) or the crinoid *Thalassometra gracilis* (Carpenter, 1888; 4.5 ng g^–1^ [65]). Transcriptome and phylogenetic analyses confirm results for *P. phosphorea* and *A. murrayi* with a clear sequence conservation of the retrieved LUCs with the *Renilla* LUC. Consistent with the literature, species such as *D. gracile* or undetermined species from the genera *Umbellula*, *Pennatula* and *Funiculina* also present evidence of the use of CTZ as substrate and a *Renilla*-like LUC as enzyme of their bioluminescent systems [15].

Our phylogenetic analysis underlines three distinctive clades of *Renilla*-like LUC. One of these clades (clade A) contains all the known sequences of anthozoan light-emitting LUCs. The other two clades (clades B and C) contain other homologous *R*Luc-like sequences, notably from non-luminous species. The related biochemical functionality and bioactivity of these B and C clade sequences are unknown and need further investigations to fully apprehend the evolution of LUC among anthozoans. Nevertheless, this clustering led to hypothesized duplications of the ancestral gene with either (i) a neo functionality as a ‘real’ functional LUC able to catalyse a bioluminescent reaction or (ii) a bi-functionality of these enzymes as light-producing enzyme and another ancestrally conserved enzymatic function. The ancestral functionality of the *R*Luc enzyme has been assumed to originate from bacterial haloalkane dehalogenases, enzymes with a hydrolase activity cleaving bonds in halogenated compounds [66]. A basal gene transfer from bacteria to metazoans has been hypothesized [18,66]. Interestingly, *R*Luc-like LUCs have been demonstrated to be the enzymes involved in the bioluminescence of phylogenetically distinct species such as the brittle star *A. filiformis* [18] and the tunicate *P. atlanticum* (Péron, 1804) [19], letting assumed that the sequence was convergently coopted multiple times during the evolution. Consistent with previous research on *R*Luc sequences, our retrieved *P. phosphorea* and *A. murrayi* LUC (clade A) present high sequence similarity with the other pennatulaceans functional LUCs, also revealing conservation of the catalytic triads essential for the LUC catalytic activity [12,51,67]. The complete characterization of *P. phosphorea*, *A. murrayi* and *F. quadrangularis* LUCs will allow us to better apprehend the functionality and evolution of these LUC enzymes. Future research will enable us to better characterize these LUCs and also the interactions between these enzymes and their substrates and associated molecules, as is the case with the approach to the role of *Renilla* LUC residues (e.g. mutagenesis, quantum mechanics/molecular mechanics methods [12,51,67]).

While the retrieved GFP sequences are unique and well clustered with other Scleralcyonacea sequences, a similar observation, as for the LUCs, occurs for the CBPs and CBPs-like with multiple retrieved sequences clustered in different groups. The first group represents the functional CBP retrieved and characterized in luminous species, while the second group, named photoprotein-like proteins, raise questions on the exact functionality of these retrieved sequences. These photoprotein-like proteins might also be involved in calcium binding. Active photoproteins of cnidarians and ctenophores depend on calcium to trigger light emission [68–71]. Nevertheless, the higher expression within the *P. phosphorea* peduncle assumed another function without a relationship with the bioluminescence.

AlphaFold is a neural network machine learning tool for predicting macromolecular structures and complexes, providing structural models with near-atomic accuracy even in the absence of known similar structures [72,73]. Here, we employed AlphaFold to predict macromolecular structures of the most expressed genes encoding for LUC, GFP and CBP proteins in *P. phosphorea* and *A. murrayi*. Computational predictions yielded structural models with very high confidence scores (>90) for all analysed proteins and that are structurally similar to crystallographic structures of well-characterized LUC [12], GFP [14] and CBP [74] proteins encoded by the sea pansies *R. reniformis* and *R. muelleri*. Taken together, our structural predictions suggest that the identified *P. phosphorea* and *A. murrayi* LUC, GFP and CBP proteins with the highest expression values are structurally and functionally relevant and responsible for bioluminescence in these species.

Kept in captivity without exogenous CTZ supply, *P. phosphorea* can still produce light after 1 year, even if the CTZ content and LUC activity decrease. Therefore, these results support a *de novo* synthesis of the CTZ substrate in *P. phosphorea*. CTZ *de novo* synthesis by luminous marine organisms has been documented for the calanoid copepods *Metridia longa* (Lubbock, 1854) and *M. pacifica* (Brodsky, 1950), the oplophorid shrimp *Systellaspis debilis* (Milne-Edwards, 1881) and two ctenophores, *Mnemiopsis leidyi* (Agassiz, 1865) and *Bolinopsis infundibulum* (Müller, 1776) [75–78]. The natural precursors of CTZ have been demonstrated to be the ʟ-tyrosine and ʟ-phenylalanine amino acids in *M. pacifica* [77]. By screening the transcriptomic data of 24 ctenophores, Francis *et al*. [79] assumed the involvement of a non-haeme iron oxidase-like enzyme, similar to isopenicillin-N-synthase, in the biosynthesis pathway of this luciferin [79]. Future research could be carried out to validate the *de novo* biosynthesis of the CTZ bioluminescent substrate, in particular by maintaining *P. phosphorea* and their offspring over generations in captivity in the same conditions without CTZ supply [78,80]. This protocol could be applied to other pennatulaceans to determine whether the CTZ genesis is a common trait in this clade. Nevertheless, the first challenge would be to control the life cycle of these species in captivity. Similarly, it would be interesting to analyse different pennatulacean transcriptomes searching for enzymes homologous to the isopenicillin-N-synthase potentially involved in the CTZ biosynthetic pathway, as retrieved in ctenophores.

In *P. phosphorea*, the morphological localization of LUC expression matched the green fluorescent sites obtained in unfixed and fixed tissues. Green autofluorescence observed on unfixed specimens is assumed to be a mix of the native autofluorescence of CTZ and the autofluorescent reaction occurring through the GFP, while green autofluorescence observed after tissue fixation corresponds only to the GFP signals. Comparatively, the *in vivo* green fluorescence observed in the *F. quadrangularis* tissues, which disappeared after fixation, was assumed to be related only to the natural autofluorescence of CTZ and the lack of GFP in this species. In contrast to *P. phosphorea,* which emits green waves of light at *λ*_max_ = 510 nm, *F. quadrangularis* emits blue at *λ*_max_ = 485 nm, supporting the absence of GFP for this species [15,36,61,81]. This natural autofluorescence has recently been demonstrated to appear and disappear from the photogenic site, depending on the substrate dietary acquisition of the brittle star *A. filiformis*. This species depends on the trophic acquisition of the CTZ substrate to produce light [64,82]. When the brittle star was fed with CTZ-containing food, green autofluorescent spots appeared at the level of spine-associated photocytes [82]. As for *F. quadrangularis*, a similar disappearance of the green fluorescent signal (possibly attributed to the CTZ) has been observed in the fixed tissue of the brittle star species [82]. The autofluorescent sites observed along the tentacle bases of the autozooids of *P. phosphorea* are consistent with the already described location of the autofluorescent photogenic cell processes in autozooids of *Stylatula elongata* [83] and, to a lesser extent, *Acanthoptilum gracile* and *Renilla koellikeri* [33,83]. For the former species, it was noticed that the photocytes process followed the same orientation as the longitudinal muscles, allowing autozooids to retract [33]. On the other hand, the LUC expression site in *F. quadrangularis*, in the cellular processes of the apical part of the tentacle, was never reported before. This location along the polyp tentacles matches the described position of photocytes in autozooids of *Ptilosarcus* species [83].

Our spectrum measurement performed with the *A. murrayi* recombinant LUC is consistent with already observed CTZ-dependent systems in other species emitting in the same range of wavelengths. Recorded spectrum for CTZ-dependent LUC systems can vary between 475 and 493 nm in *P. atlanticum*, 482 nm emitted by *R*Luc, 472 nm emitted by the brittle star *A. filiformis*, 455 nm emitted by *Oplophorus gracilirostris* and 485 nm emitted by *F. quadrangularis* [18,19,66,84]. According to the natural spectrum recorded on the whole specimens (*λ*_max_ = 513 nm), similar to the spectrum measured for *P. phosphorea* [61], and the transcriptomic presence of a GFP sequence in *A. murrayi*, this species is strongly assumed to display a GFP-associated CTZ-dependent luminous system. At least six anthozoan species present expression of a GFP and the production of green light. Some of these species may live in sympatry with anthozoan blue emitters, such as *P. phosphorea* and *F. quadrangularis,* with an overlap of depth repartition and habitat preferences occurring. Therefore, questions arise concerning luminescence’s exact function(s) among anthozoans. Even if some assumptions are proposed in the literature, no one has ever developed an ethological protocol to validate them [37]. Therefore, the question remains as to why some anthozoans evolved green light emission while the primary CTZ–LUC reaction produces blue light. Different function(s) in the same environment may have led to the acquisition or the loss of the GFP gene by some anthozoan species upon evolutionary constraints.

As demonstrated for other pennatulacean species, CBP seems to be an essential component of the luminous system [21–25,27]. The retrieved CBPs in *P. phosphorea* and *A. murrayi* are congruent with this literature. GO distribution analyses performed on the different tissues of *P. phosphorea* underline a high expression of calcium ion-binding proteins, including the retrieved CL1333 CBP, in the photogenic tissue (pinnule and rachis) of this species. The expression of this gene within the photogenic tissues supports its involvement in the luminous reaction. As demonstrated for *Renilla* by Stepanyuk *et al*. [29], CBPs need calcium as a co-factor to release the CTZ. In addition to the retrieved CBP sequence in the *Pennatula* transcriptome, our calcium assay results highlight the involvement of the calcium ion in the light-emission process of both *P. phosphorea* and *F. quadrangularis*. Results obtained for the calcium ionophore A23187 reveal that the ion action does not result from intracellular calcium storage but rather is provided by external calcium input. Moreover, calcium is shown to be essential for the physiological luminescent response through adrenaline application. Pieces of evidence of calcium involvement in anthozoan luminescence were already described for *R. reniformis* and *V. cynomorium* [27,28].

These results let us assume a conservation of the CTZ-dependent *Renilla*-like LUC bioluminescent system, involving also a CBP, across luminous pennatulaceans (electronic supplementary material, table S1) [8]. The involvement of GFP appears species-dependent, with only a few species emitting blue light (electronic supplementary material, table S1) [8]. Nevertheless, deeper investigations are needed to fully apprehend the conservation of those actors across the diversity of luminous pennatulaceans.

**Figure 9.**
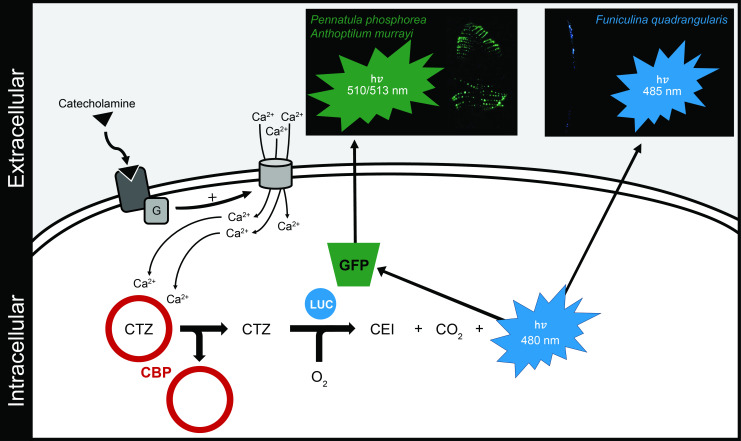
Schematic of the putative pathway driving the luminescence production in sea pens. Elements of this pathway have been compiled from the present results and the literature [12,15,17,21–25,32–36]. CBP, coelenterazine-binding protein; CEI, coelenteramide; CTZ, coelenterazine; G, G protein; GFP, green fluorescent protein; LUC, luciferase. The clear colocalization of specific molecular actors (e.g. catecholaminergic receptors, CBP, GFP, LUC) still needs to be established to confirm the following predicted pathway.

A hypothetical scheme of the generalized pathway could be established using our results and the literature on the pennatulacean luminescence mechanism (figure 9). The first step is the activation of catecholaminergic receptors through the binding of biogenic amines (mainly adrenaline and noradrenaline [35,36]), which will release an intracellular-associated G protein [85,86]. G protein could be involved in a large variety of intracellular pathways [87], some of which involve increasing intracellular calcium (via direct or indirect activation of calcium channels) [88–91]. This intracellular calcium increase will lead to the release of the CTZ through the binding of this ion on the CBP, leaving this luciferin free to react with LUC in the presence of oxygen to produce blue light around 480 nm [9–11,13,21–25,27]. For pennatulaceans lacking GFP (e.g. *F. quadrangularis*), this scheme ends here with the direct emission in blue colour, while for those displaying GFP expression, the blue light is captured by this specific fluorescent protein and re-emitted in green wavelength [22], such as for *P. phosphorea*. Future research is needed to validate this hypothetical scheme, and further investigations will be conducted to establish the functional activities of all these components in less-studied sea pens. Astonishingly, even if attempts were made on other anthozoans over the past decades, the *Renilla* bioluminescence system remains the only isolated and cloned system [12,13,22–25,27]. Despite the demonstrated widespread uses of the sea pansy bioluminescent system in biotechnology and biomedicine (e.g. [92–94]), the *Renilla* LUC stands as the only one of the most commercially employed gene reporters in biomolecular sciences. Nonetheless, in the *Renilla* bioluminescence system, the exact action mode of CBP and calcium is not fully apprehended [29,95]. Considering these facts, our introspection into the bioluminescent system of other luminous pennatulaceans could be of great use for new biotechnological advances. Our results provided a better understanding of the evolution of the bioluminescence system and associated molecules from these enigmatic benthic sessile organisms.

## Data Availability

Transcriptome raw reads were uploaded as Sequence Reads Archives (SRA): *A. murrayi* (PRJNA1144931), *P. phosphorea* (PRJNA1152785). The unigene annotation tables are accessible from the corresponding author upon request. Supplementary material is available online [96].

## References

[1] Shimomura O, Yampolsky I. 2019 Bioluminescence: chemical principles and methods, 3rd edn. Singapore: World Scientific Publishing.

[2] Haddock S, Moline M, Case J. 2010 Bioluminescence in the sea. Annu. Rev. Mar. Sci **2**, 443–493. (10.1146/annurev-marine-120308-081028) 21141672

[3] Delroisse J, Duchatelet L, Flammang P, Mallefet J. 2021 Leaving the dark side? Insights into the evolution of luciferases. Front. Mar. Sci. **8**, 673620. (10.3389/fmars.2021.673620)

[4] Fleiss A, Sarkisyan KS. 2019 A brief review of bioluminescent systems (2019). Curr. Genet. **65**, 877–882. (10.1007/s00294-019-00951-5) 30850867 PMC6620254

[5] Hastings JW. 1996 Chemistries and colors of bioluminescent reactions: a review. Gene **173**, 5–11. (10.1016/0378-1119(95)00676-1) 8707056

[6] Wilson T, Hastings J. 2012 Bioluminescence: living lights, lights for living. Cambridge, MA: Harvard University Press.

[7] Kaskova ZM, Tsarkova AS, Yampolsky IV. 2016 1001 lights: luciferins, luciferases, their mechanisms of action and applications in chemical analysis, biology and medicine. Chem. Soc. Rev. **45**, 6048–6077. (10.1039/c6cs00296j) 27711774

[8] Duchatelet L, Dupont S. 2024 Marine eukaryote bioluminescence: a review of species and their functional biology. Mar. Life Sci. Technol. (10.1007/s42995-024-00250-0) PMC12102453 40417256

[9] Cormier MJ. 1960 Studies of the bioluminescence of Renilla reniformis. Biochim. Biophys. Acta **42**, 333–343. (10.1016/0006-3002(60)90797-6) 13695546

[10] Matthews JC, Hori K, Cormier MJ. 1977 Purification and properties of Renilla reniformis luciferase. Biochemistry **16**, 85–91. (10.1021/bi00620a014) 12797

[11] Lorenz WW, McCann RO, Longiaru M, Cormier MJ. 1991 Isolation and expression of a cDNA encoding Renilla reniformis luciferase. Proc. Natl Acad. Sci. USA **88**, 4438–4442. (10.1073/pnas.88.10.4438) 1674607 PMC51675

[12] Schenkmayerova A *et al*. 2023 Catalytic mechanism for Renilla-type luciferases. Nat. Catal. **6**, 23–38. (10.1038/s41929-022-00895-z)

[13] Inoue S, Kakoi H, Murata M, Goto T, Shimomura O. 1977 Complete structure of Renilla luciferin and luciferyl sulfate. Tetrahedron Lett. **18**, 2685–2688. (10.1016/s0040-4039(01)83046-x)

[14] Loening AM, Fenn TD, Gambhir SS. 2007 Crystal structures of the luciferase and green fluorescent protein from Renilla reniformis. J. Mol. Biol. **374**, 1017–1028. (10.1016/j.jmb.2007.09.078) 17980388 PMC2700051

[15] Bessho-Uehara M, Francis WR, Haddock SHD. 2020 Biochemical characterization of diverse deep-sea anthozoan bioluminescence systems. Mar. Biol. **167**, 114. (10.1007/s00227-020-03706-w)

[16] DeLeo DM, Bessho-Uehara M, Haddock SHD, McFadden CS, Quattrini AM. 2024 Evolution of bioluminescence in anthozoa with emphasis on octocorallia. Proc. R. Soc. B **291**, 20232626. (10.1098/rspb.2023.2626) PMC11040251 38654652

[17] Cormier MJ, Hori K, Karkhanis YD, Anderson JM, Wampler JE, Morin JG, Hastings JW. 1973 Evidence for similar biochemical requirements for bioluminescence among the coelenterates. J. Cell. Physiol. **81**, 291–297. (10.1002/jcp.1040810218) 4144397

[18] Delroisse J, Ullrich-Lüter E, Blaue S, Ortega-Martinez O, Eeckhaut I, Flammang P, Mallefet J. 2017 A puzzling homology: a brittle star using a putative cnidarian-type luciferase for bioluminescence. Open Biol. **7**, 160300. (10.1098/rsob.160300) 28381628 PMC5413902

[19] Tessler M *et al*. 2020 A putative chordate luciferase from a cosmopolitan tunicate indicates convergent bioluminescence evolution across phyla. Sci. Rep. **10**, 17724. (10.1038/s41598-020-73446-w) 33082360 PMC7576829

[20] Lau ES *et al*. 2024 Functional characterization of luciferase in a brittle star indicates parallel evolution influenced by genomic availability of haloalkane dehalogenase. BioRxiv. (10.1101/2024.10.14.618359) PMC12059646 40181585

[21] Anderson J, Charbonneau H, Cormier M. 1974 Mechanism of calcium induction of Renilla bioluminescence. Involvement of a calcium-triggered luciferin binding protein. Biochemistry **13**, 1195–1200. (10.1021/bi00703a602) 4149963

[22] Ward WW, Cormier MJ. 1979 An energy transfer protein in coelenterate bioluminescence. Characterization of the Renilla green-fluorescent protein. J. Biol. Chem. **254**, 781–788. (10.1016/s0021-9258(17)37873-0) 33175

[23] Kumar S, Harrylock M, Walsh KA, Cormier MJ, Charbonneau H. 1990 Amino acid sequence of the Ca^2+^-triggered luciferin binding protein of Renilla reniformis. FEBS Lett. **268**, 287–290. (10.1016/0014-5793(90)81029-n) 1974522

[24] Inouye S. 2007 Expression, purification and characterization of calcium-triggered luciferin-binding protein of Renilla reniformis. Protein Expr. Purif. **52**, 66–73. (10.1016/j.pep.2006.07.028) 16997571

[25] Titushin MS, Markova SV, Frank LA, Malikova NP, Stepanyuk GA, Lee J, Vysotski ES. 2008 Coelenterazine-binding protein of Renilla muelleri: cDNA cloning, overexpression, and characterization as a substrate of luciferase. Photochem. Photobiol. Sci. **7**, 189–196. (10.1039/b713109g) 18264586

[26] Ogoh K, Kinebuchi T, Murai M, Takahashi T, Ohmiya Y, Suzuki H. 2013 Dual‐color‐emitting green fluorescent protein from the sea cactus Cavernularia obesa and its use as a pH indicator for fluorescence microscopy. Luminescence **28**, 582–591. (10.1002/bio.2497) 23468077 PMC3884763

[27] Henry JP, Ninio M. 1978 Control of the Ca^2+^-triggered bioluminescence of Veretillum cynomorium lumisomes. Biochim. Biophys. Acta **504**, 40–59. (10.1016/0005-2728(78)90005-1) 30480

[28] Charbonneau H, Cormier MJ. 1979 Ca^2+^-induced bioluminescence in Renilla reniformis. Purification and characterization of a calcium-triggered luciferin-binding protein. J. Biol. Chem. **254**, 769–780. (10.1016/s0021-9258(17)37872-9) 33174

[29] Stepanyuk GA, Liu ZJ, Vysotski ES, Lee J, Rose JP, Wang BC. 2009 Structure based mechanism of the Ca^2+^‐induced release of coelenterazine from the Renilla binding protein. Proteins **74**, 583–593. (10.1002/prot.22173) 18655070

[30] Nicol JAC. 1955 Observations on luminescence in Renilla (Pennatulacea). J. Exp. Biol. **32**, 32. (10.1242/jeb.32.2.299)

[31] Davenport D, Nicol J. 1956 Observations on luminescence in sea pens (Pennatulacea). Proc. R. Soc. B **144**, 480–496. (10.1098/rspb.1956.0005)

[32] Wampler JE, Karkhanis YD, Morin JG, Cormier MJ. 1973 Similarities in the bioluminescence from the Pennatulacea. Biochim. Biophys. Acta **314**, 104–109. (10.1016/0005-2728(73)90068-6) 4147478

[33] Satterlie RA, Anderson PAV, Case JF. 1980 Colonial coordination in anthozoans: Pennatulacea. Mar. Behav. Physiol. **7**, 25–46. (10.1080/10236248009386969)

[34] Germain G, Anctil M. 1988 Luminescent activity and ultrastructural characterization of photocytes dissociated from the coelenterate Renilla köllikeri. Tissue Cell **20**, 701–720. (10.1016/0040-8166(88)90017-1) 18620241

[35] Anctil M, Boulay D, Larivière L. 1982 Monoaminergic mechanisms associated with control of luminescence and contractile activities in the coelenterate, Renilla köllikeri. J. Exp. Zool. **223**, 11–24. (10.1002/jez.1402230103)

[36] Duchatelet L, Coubris C, Pels C, Dupont ST, Mallefet J. 2023 Catecholamine involvement in the bioluminescence control of two species of anthozoans. Life **13**, 1798. (10.3390/life13091798) 37763202 PMC10533100

[37] Morin JG. 1976 Probable functions of bioluminescence in the Pennatulacea (Cnidaria, Anthozoa). In Coelenterate ecology and behavior (ed. GO Mackie), pp. 629–638. Boston, MA: Springer. (10.1007/978-1-4757-9724-4_65)

[38] Delroisse J, Mallefet J, Flammang P. 2016 De novo adult transcriptomes of two European brittle stars: spotlight on opsin-based photoreception. PLoS ONE **11**, e0152988. (10.1371/journal.pone.0152988) 27119739 PMC4847921

[39] Delroisse J, Duchatelet L, Flammang P, Mallefet J. 2018 De novo transcriptome analyses provide insights into opsin-based photoreception in the lanternshark Etmopterus spinax. PLoS ONE **13**, e0209767. (10.1371/journal.pone.0209767) 30596723 PMC6312339

[40] Delroisse J, Duchatelet L, Flammang P, Mallefet J. 2021 Photophore distribution and enzymatic diversity within the photogenic integument of the cookie-cutter shark Isistius brasiliensis (Chondrichthyes: Dalatiidae). Front. Mar. Sci. **8**, 627045. (10.3389/fmars.2021.627045)

[41] Cock PJA, Fields CJ, Goto N, Heuer ML, Rice PM. 2010 The Sanger FASTQ file format for sequences with quality scores, and the Solexa/Illumina FASTQ variants. Nucleic Acids Res. **38**, 1767–1771. (10.1093/nar/gkp1137) 20015970 PMC2847217

[42] Grabherr MG *et al*. 2011 Full-length transcriptome assembly from RNA-Seq data without a reference genome. Nat. Biotechnol. **29**, 644–652. (10.1038/nbt.1883) 21572440 PMC3571712

[43] Pertea G *et al*. 2003 TIGR gene indices clustering tools (TGICL): a software system for fast clustering of large EST datasets. Bioinformatics **19**, 651–652. (10.1093/bioinformatics/btg034) 12651724

[44] Das S, Shyamal S, Durica DS. 2016 Analysis of annotation and differential expression methods used in RNA-seq studies in crustacean systems. Integr. Comp. Biol. **56**, 1067–1079. (10.1093/icb/icw117) 27940611

[45] Altschul SF, Gish W, Miller W, Myers EW, Lipman DJ. 1990 Basic local alignment search tool. J. Mol. Biol. **215**, 403–410. (10.1016/s0022-2836(05)80360-2) 2231712

[46] Buchfink B, Xie C, Huson DH. 2014 Fast and sensitive protein alignment using DIAMOND. Nat. Methods **12**, 59–60. (10.1038/nmeth.3176) 25402007

[47] Conesa A, Götz S, García-Gómez JM, Terol J, Talón M, Robles M. 2005 Blast2GO: a universal tool for annotation, visualization and analysis in functional genomics research. Bioinformatics **21**, 3674–3676. (10.1093/bioinformatics/bti610) 16081474

[48] Quevillon E, Silventoinen V, Pillai S, Harte N, Mulder N, Apweiler R, Lopez R. 2005 InterProScan: protein domains identifier. Nucleic Acids Res. **33**, W116–W120. (10.1093/nar/gki442) 15980438 PMC1160203

[49] Haas BJ *et al*. 2013 De novo transcript sequence reconstruction from RNA-seq using the Trinity platform for reference generation and analysis. Nat. Protoc. **8**, 1494–1512. (10.1038/nprot.2013.084) 23845962 PMC3875132

[50] Kearse M *et al*. 2012 Geneious Basic: an integrated and extendable desktop software platform for the organization and analysis of sequence data. Bioinformatics **28**, 1647–1649. (10.1093/bioinformatics/bts199) 22543367 PMC3371832

[51] Woo J, Howell MH, von Arnim AG. 2009 Structure–function studies on the active site of the coelenterazine‐dependent luciferase from Renilla. Protein Sci. **17**, 725–735. (10.1110/ps.073355508) PMC2271170 18359861

[52] McFadden CS, Ofwegen LP, Quattrini AM. 2022 Revisionary systematics of Octocorallia (Cnidaria: Anthozoa) guided by phylogenomics. Bull. Soc. Syst. Biol. **1**, 8735. (10.18061/bssb.v1i3.8735)

[53] Labas YA, Gurskaya NG, Yanushevich YG, Fradkov AF, Lukyanov KA, Lukyanov SA, Matz MV. 2002 Diversity and evolution of the green fluorescent protein family. Proc. Natl Acad. Sci. USA **99**, 4256–4261. (10.1073/pnas.062552299) 11929996 PMC123635

[54] Alieva NO *et al*. 2008 Diversity and evolution of coral fluorescent proteins. PLoS ONE **3**, e2680. (10.1371/journal.pone.0002680) 18648549 PMC2481297

[55] Li G, Zhang QJ, Zhong J, Wang YQ. 2009 Evolutionary and functional diversity of green fluorescent proteins in cephalochordates. Gene **446**, 41–49. (10.1016/j.gene.2009.07.003) 19615432

[56] Shagin DA *et al*. 2004 GFP-like proteins as ubiquitous metazoan superfamily: evolution of functional features and structural complexity. Mol. Biol. Evol. **21**, 841–850. (10.1093/molbev/msh079) 14963095

[57] Schnitzler CE *et al*. 2012 Genomic organization, evolution, and expression of photoprotein and opsin genes in Mnemiopsis leidyi: a new view of ctenophore photocytes. BMC Biol. **10**, 107. (10.1186/1741-7007-10-107) 23259493 PMC3570280

[58] Capella-Gutiérrez S, Silla-Martínez JM, Gabaldón T. 2009 trimAl: a tool for automated alignment trimming in large-scale phylogenetic analyses. Bioinformatics **25**, 1972–1973. (10.1093/bioinformatics/btp348) 19505945 PMC2712344

[59] Nguyen LT, Schmidt HA, von Haeseler A, Minh BQ. 2015 IQ-TREE: a fast and effective stochastic algorithm for estimating maximum-likelihood phylogenies. Mol. Biol. Evol. **32**, 268–274. (10.1093/molbev/msu300) 25371430 PMC4271533

[60] Posada D, Crandall KA. 1998 MODELTEST: testing the model of DNA substitution. Bioinformatics **14**, 817–818. (10.1093/bioinformatics/14.9.817) 9918953

[61] Nicol J. 1958 Observations on the luminescence of Pennatula phosphorea, with a note on the luminescence of Virgularia mirabilis. J. Mar. Biol. Assoc. UK **37**, 551–563. (10.1017/S0025315400005610)

[62] Rahnama S, Saffar B, Kahrani ZF, Nazari M, Emamzadeh R. 2017 Super RLuc8: a novel engineered Renilla luciferase with a red-shifted spectrum and stable light emission. Enzym. Microb. Technol. **96**, 60–66. (10.1016/j.enzmictec.2016.09.009) 27871386

[63] Khoshnevisan G, Emamzadeh R, Nazari M, Rasa SMM, Sariri R, Hassani L. 2018 Kinetics, structure, and dynamics of Renilla luciferase solvated in binary mixtures of glycerol and water and the mechanism by which glycerol obstructs the enzyme emitter site. Int. J. Biol. Macromol. **117**, 617–624. (10.1016/j.ijbiomac.2018.05.160) 29800661

[64] Mallefet J, Duchatelet L, Coubris C. 2020 Bioluminescence induction in the ophiuroid Amphiura filiformis (Echinodermata). J. Exp. Biol. **223**, jeb218719. (10.1242/jeb.218719) 31974222

[65] Mallefet J, Martinez-Soares P, Eléaume M, O’Hara T, Duchatelet L. 2023 New insights on crinoid (Echinodermata; Crinoidea) bioluminescence. Front. Mar. Sci. **10**, 1136138. (10.3389/fmars.2023.1136138)

[66] Loening AM. 2006 Consensus guided mutagenesis of Renilla luciferase yields enhanced stability and light output. Protein Eng. Des. Sel. **19**, 391–400. (10.1093/protein/gzl023) 16857694

[67] Nandi A, Zhang A, Chu ZT, Xie WJ, Xu Z, Dong S, Warshel A. 2024 Exploring the light-emitting agents in Renilla luciferases by an effective QM/MM approach. J. Am. Chem. Soc. **146**, 13875–13885. (10.1021/jacs.4c00963) 38718165 PMC11293844

[68] Tsuji FI, Ohmiya Y, Fagan TF, Toh H, Inouye S. 1995 Molecular evolution of the Ca^2+^‐binding photoproteins of the Hydrozoa. Photochem. Photobiol. **62**, 657–661. (10.1111/j.1751-1097.1995.tb08713.x) 7480150

[69] Powers ML, McDermott AG, Shaner NC, Haddock SHD. 2013 Erratum to ‘Expression and characterization of the calcium-activated photoprotein from the ctenophore Bathocyroe fosteri: insights into light-sensitive photoproteins’. Biochem. Biophys. Res. Commun. **435**, 751. (10.1016/j.bbrc.2008.07.166) PMC3570696 23262181

[70] Burakova LP, Stepanyuk GA, Eremeeva EV, Vysotski ES. 2016 Role of certain amino acid residues of the coelenterazine-binding cavity in bioluminescence of light-sensitive Ca^2+^-regulated photoprotein berovin. Photochem. Photobiol. Sci. **15**, 691–704. (10.1039/c6pp00050a) 27117544

[71] Burakova LP, Vysotski ES. 2019 Recombinant Ca^2+^-regulated photoproteins of ctenophores: current knowledge and application prospects. Appl. Microbiol. Biotechnol. **103**, 5929–5946. (10.1007/s00253-019-09939-0) 31172204

[72] Jumper J *et al*. 2021 Highly accurate protein structure prediction with AlphaFold. Nature **596**, 583–589. (10.1038/s41586-021-03819-2) 34265844 PMC8371605

[73] Abramson J *et al*. 2024 Accurate structure prediction of biomolecular interactions with AlphaFold 3. Nature **630**, 493–500. (10.1038/s41586-024-07487-w) 38718835 PMC11168924

[74] Stepanyuk GA, Liu ZJ, Markova SS, Frank LA, Lee J, Vysotski ES, Wang BC. 2008 Crystal structure of coelenterazine-binding protein from Renilla muelleri at 1.7 Å: why it is not a calcium-regulated photoprotein. Photochem. Photobiol. Sci. **7**, 442–447. (10.1039/b716535h) 18385886

[75] Buskey EJ, Stearns DE. 1991 The effects of starvation on bioluminescence potential and egg release of the copepod Metridia longa. J. Plankton Res. **13**, 885–893. (10.1093/plankt/13.4.885)

[76] Thomson CM, Herring PJ, Campbell AK. 1995 Evidence for de novo biosynthesis of coelenterazine in the bioluminescent midwater shrimp, Systellaspis debilis C. J. Mar. Biol. Assoc. UK **75**, 165–171. (10.1017/s0025315400015277)

[77] Oba Y, Kato S ichi, Ojika M, Inouye S. 2009 Biosynthesis of coelenterazine in the deep-sea copepod, Metridia pacifica. Biochem. Biophys. Res. Commun. **390**, 684–688. (10.1016/j.bbrc.2009.10.028) 19833098

[78] Bessho-Uehara M, Huang W, Patry WL, Browne WE, Weng JK, Haddock SHD. 2020 Evidence for de novo biosynthesis of the luminous substrate coelenterazine in ctenophores. iScience **23**, 101859. (10.1016/j.isci.2020.101859) 33376974 PMC7756133

[79] Francis WR, Shaner NC, Christianson LM, Powers ML, Haddock SHD. 2015 Occurrence of isopenicillin-N-synthase homologs in bioluminescent ctenophores and implications for coelenterazine biosynthesis. PLoS ONE **10**, e0128742. (10.1371/journal.pone.0128742) 26125183 PMC4488382

[80] Haddock SH, Rivers TJ, Robison BH. 2001 Can coelenterates make coelenterazine? Dietary requirement for luciferin in cnidarian bioluminescence. Proc. Natl Acad. Sci. USA **98**, 11148–11151. (10.1073/pnas.201329798) 11572972 PMC58698

[81] Herring PJ. 1991 Observations on bioluminescence in some deep-water anthozoans. In Coelenterate biology: recent research on Cnidaria and Ctenophora (eds RB Williams, PFS Cornelius, RG Hughes, EA Robson), pp. 573–579. Dordrecht, The Netherlands: Springer. (10.1007/978-94-011-3240-4_80)

[82] Coubris C, Duchatelet L, Delroisse J, Bayaert WS, Parise L, Eloy MC, Pels C, Mallefet J. 2024 Maintain the light, long-term seasonal monitoring of luminous capabilities in the brittle star Amphiura filiformis. Sci. Rep. **14**, 13238. (10.1038/s41598-024-64010-x) 38853171 PMC11163003

[83] Herring PJ. 1978 Bioluminescence of invertebrates other than insects. In Bioluminescence in action (ed PJ Herring), pp. 199–240. London, UK: Academic Press.

[84] Inouye S, Watanabe K, Nakamura H, Shimomura O. 2000 Secretional luciferase of the luminous shrimp Oplophorus gracilirostris: cDNA cloning of a novel imidazopyrazinone luciferase. FEBS Lett. **481**, 19–25. (10.1016/s0014-5793(00)01963-3) 10984608

[85] Kobilka B. 1992 Adrenergic receptors as models for G protein-coupled receptors. Annu. Rev. Neurosci. **15**, 87–114. (10.1146/annurev.ne.15.030192.000511) 1575451

[86] Caron MG, Lefkowitz RJ. 1993 Catecholamine receptors: structure, function, and regulation. Recent Prog. Horm. Res. **1993**, 277–290. (10.1016/b978-0-12-571148-7.50014-2) 8441851

[87] Neves SR, Ram PT, Iyengar R. 2002 G protein pathways. Science **296**, 1636–1639. (10.1126/science.1071550) 12040175

[88] Brown AM, Birnbaumer L. 1988 Direct G protein gating of ion channels. Am. J. Physiol. Heart Circ. Physiol. **254**, H401–H410. (10.1152/ajpheart.1988.254.3.h401) 2450476

[89] Hille B. 1994 Modulation of ion-channel function by G-protein-coupled receptors. Trends Neurosci. **17**, 531–536. (10.1016/0166-2236(94)90157-0) 7532338

[90] Dolphin AC. 2003 G protein modulation of voltage-gated calcium channels. Pharmacol. Rev. **55**, 607–627. (10.1124/pr.55.4.3) 14657419

[91] Tedford HW, Zamponi GW. 2006 Direct G protein modulation of Cav2 calcium channels. Pharmacol. Rev. **58**, 837–862. (10.1124/pr.58.4.11) 17132857

[92] Roda A, Pasini P, Mirasoli M, Michelini E, Guardigli M. 2004 Biotechnological applications of bioluminescence and chemiluminescence. Trends Biotechnol. **22**, 295–303. (10.1016/j.tibtech.2004.03.011) 15158059

[93] Kirkpatrick A, Xu T, Ripp S, Sayler G, Close D. 2019 Biotechnological advances in luciferase enzymes. In Bioluminescence: analytical applications and basic biology (ed. S Hirobumi), pp. 1–23. London, UK: IntechOpen. (10.5772/intechopen.85313)

[94] Welsh DK, Kay SA. 2005 Bioluminescence imaging in living organisms. Curr. Opin. Biotechnol. **16**, 73–78. (10.1016/j.copbio.2004.12.006) 15722018

[95] Kudryavtsev AN, Krasitskaya VV, Efremov MK, Zangeeva SV, Rogova AV, Tomilin FN, Frank LA. 2023 Ca^2+^-triggered coelenterazine-binding protein Renilla expected and unexpected features. Int. J. Mol. Sci. **24**, 2144. (10.3390/ijms24032144) 36768474 PMC9917264

[96] Duchatelet L, Galeazzo GA, Coubris C, Bridoux L, Rezsohazy R, Melo MRS *et al*. 2025 Supplementary material from: Insights into the bioluminescence systems of three sea pens (Cnidaria: Anthozoa): from de novo transcriptome analyses to biochemical assays. Figshare. (10.6084/m9.figshare.c.7735482) PMC12040472 40300651

